# Proteomic Studies of Roots in Hypoxia-Sensitive and -Tolerant Tomato Accessions Reveal Candidate Proteins Associated with Stress Priming

**DOI:** 10.3390/cells11030500

**Published:** 2022-01-31

**Authors:** Małgorzata Czernicka, Kinga Kęska, Sébastien Planchon, Małgorzata Kapusta, Marzena Popielarska-Konieczna, Wojciech Wesołowski, Marek Szklarczyk, Jenny Renaut

**Affiliations:** 1 Department of Plant Biology and Biotechnology, Faculty of Biotechnology and Horticulture, University of Agriculture in Krakow, Al. 29 Listopada 54, 31-425 Kraków, Poland; k.keska@op.pl (K.K.); w.wesolowski@urk.edu.pl (W.W.); m.szklarczyk@urk.edu.pl (M.S.); 2 Environmental Research and Innovation Department, Luxembourg Institute of Science and Technology, 41 Rue du Brill, L-4422 Belvaux, Luxembourg; sebastien.planchon@list.lu (S.P.); jenny.renaut@list.lu (J.R.); 3 Department of Plant Cytology and Embryology, University of Gdańsk, 59 Wita Stwosza, 80-308 Gdańsk, Poland; malgorzata.kapusta@biol.ug.edu.pl; 4 Department of Plant Cytology and Embryology, Institute of Botany, Faculty of Biology, The Jagiellonian University in Kraków, Gronostajowa 9, 30-387 Krakow, Poland; m.popielarska-konieczna@uj.edu.pl

**Keywords:** alcohol dehydrogenase, *adh2* gene expression, ADH immunodetection and immunolocalization, 2D DIGE, hypoxia, glyceraldehyde-3-phosphate dehydrogenase, MALDI TOF/TOF, priming, *Solanum lycopersicum* L., waterlogging

## Abstract

Tomato (*Solanum lycopersicum* L.) is a vegetable frequently exposed to hypoxia stress induced either by being submerged, flooded or provided with limited oxygen in hydroponic cultivation systems. The purpose of the study was to establish the metabolic mechanisms responsible for overcoming hypoxia in two tomato accessions with different tolerance to this stress, selected based on morphological and physiological parameters. For this purpose, 3-week-old plants (plants at the juvenile stage) of waterlogging-tolerant (WL-T), i.e., POL 7/15, and waterlogging-sensitive (WL-S), i.e., PZ 215, accessions were exposed to hypoxia stress (waterlogging) for 7 days, then the plants were allowed to recover for 14 days, after which another 7 days of hypoxia treatment was applied. Root samples were collected at the end of each time-point and 2D-DIGE with MALDI TOF/TOF, and expression analyses of gene and protein-encoded alcohol dehydrogenase (ADH2) and immunolabelling of ADH were conducted. After collating the obtained results, the different responses to hypoxia stress in the selected tomato accessions were observed. Both the WL-S and WL-T tomato accessions revealed a high amount of ADH2, which indicates an intensive alcohol fermentation pathway during the first exposure to hypoxia. In comparison to the tolerant one, the expression of the *adh2* gene was about two times higher for the sensitive tomato. Immunohistochemical analysis confirmed the presence of ADH in the parenchyma cells of the cortex and vascular tissue. During the second hypoxia stress, the sensitive accession showed a decreased accumulation of ADH protein and similar expression of the *adh2* gene in comparison to the tolerant accession. Additionally, the proteome showed a greater protein abundance of glyceraldehyde-3-phosphate dehydrogenase in primed WL-S tomato. This could suggest that the sensitive tomato overcomes the oxygen limitation and adapts by reducing alcohol fermentation, which is toxic to plants because of the production of ethanol, and by enhancing glycolysis. Proteins detected in abundance in the sensitive accession are proposed as crucial factors for hypoxia stress priming and their function in hypoxia tolerance is discussed.

## 1. Introduction

Agriculture around the world is vulnerable to adverse weather events connected with climate change, characterized by extreme temperatures and intensive rainfall. This causes abiotic stresses, such as flooding or waterlogging, which affect the plants. Waterlogging stress occurs because of heavy rainfall and inadequate soil drainage. Flooded soil leads to a decrease in oxygen concentration around the plant roots. Due to their sessile nature, plants are not able to escape from any stress factor, including waterlogging. Moreover, plants are attached firmly to the ground through their roots, where root apices are the first zones which respond to a decrease in oxygen [1]. The survival of waterlogged plants depends on acclimatization to hypoxia (low oxygen conditions) in the flooded roots. Plants respond to hypoxia stress by mediating changes in their morphology and metabolism. Indeed, because aerobic respiration is inhibited, therefore the anaerobic respiration cascade must be promoted. Energy production through oxidative phosphorylation in mitochondria has a negative effect; however, partial compensation of energy is achieved by enhancing processes such as fermentation [2], amino acid metabolism [3], the glyoxylate cycle, and the TCA cycle [4]. Glyceraldehyde-3-phosphate dehydrogenase (GAPDH) is involved in a critical energetic step of glycolysis, i.e., a process which does not require oxygen, and when glucose is converted into pyruvic acid with the formation of high-energy molecules [1]. Additionally, to survive waterlogging, plants must regenerate NADP+ and NAD+, the molecules needed for glycolysis. This is made possible via the alcohol fermentation pathway and the reduction of acetaldehyde to ethanol catalyzed with alcohol dehydrogenase (ADH) [1,5]. ADH plays a central role in anaerobic metabolism and has been observed to be one of the proteins synthesized under hypoxia conditions in maize and rice [6].

Most of the research on waterlogging stress tolerance is focused on morphological, physiological, biochemical, and molecular characterizations of cultivars. One crucial issue is discovering the key genes regulating waterlogging tolerance and impacting stress memory. Some studies have shown that plants are able to adjust to short-term hypoxia by reconfiguration of metabolic processes in roots [7]. Nevertheless, plant responses upon recovery and the reoxygenation process, or priming, have been less examined than those conferring tolerance during the stress period [1,8]. The analysis of response of root in wheat [9] and in oat [10] revealed that the recovering differed between genotypes and the physiological damages could be stronger in root tissue than in shoots. Priming could be defined as the process which occurs after pre-exposure of plants to a stress factor, resulting in “stress memory”, i.e., preparing plants to respond to secondary stress events. Although roots are the first organ to sense hypoxia under waterlogging conditions, the priming mechanisms of these organs are unknown [11]. 

The high intensity of transcription and production of mRNA under hypoxia conditions is not correlated with translation [12]. Post-transcriptional regulations concern the processing of pre-mRNA, mRNA stabilization, and finally mRNA translation for successful protein synthesis. In all likelihood, the reduction in translation is the way to save energy in order to maintain protein production. Interestingly, this rule does not concern marker genes for hypoxia, such as ADH [12]. Thus, proteomic analysis is the best technique to observe protein presence in plants under hypoxia stress. The vast majority of proteins require glycosylation or other post-translational modification processes for protein folding, stability, and signal transduction, which processes might have changed under flooding stress [13].

Tomato plants are considered to be susceptible to hypoxia stress [14]. There are reports concerning several adaptations at morphological, histological, physiological, and molecular levels in order to minimize the harmful effects of hypoxia in roots [7,14,15]. The effectiveness of adaptation to low oxygen conditions is strongly influenced by the accession, as stated also for cucumber and rice [16,17], as well as for other stress factors in tomatoes, such as chilling [18]. The analysis of the photosynthetic apparatus is used to assess the effect of different stresses, including waterlogging stress [19]. The level of decreasing in chlorophyll fluorescence in the green part of the plant could be useful for the evaluation of hypoxia stress and selection of sensitive and tolerant genotypes. In this paper, we used two tomato accessions which significantly differ in tolerance to waterlogging stress. The tomato accession POL 7/15 is described as tolerant, whereas PZ 215 is more sensitive [17]. The differences in response to waterlogging stress in selected tomato accessions were found in morphological and physiological characteristics (Supplementary Table S1). In POL 7/15, there were only significant differences in the weight and height of plants between the control and stressed plants. In the case of PZ 215, significant decreases in Fv/F_0_ (ratio of the photochemical and non-photochemical processes in photosystem II), Fv/Fm (the maximum quantum yield of PSII photochemistry), Area (area above the OJIP transient and Fm line), PI ABS (performance index on an absorption basis), ET_0_/ABS (quantum yield of electron transport from QA), and ET_0_/TR_0_ (efficiency with which a PSII trapped electron is transferred from QA) parameters, as well as increases in DI_0_/RC (the flux of energy dissipated in processes other than trapping per active PSII reaction center).

The main objective was to perform proteome analysis to identify proteins regulated under long-term waterlogging exposure in both POL 7/15 and PZ 215 tomato accessions. Special attention was paid to the expression and detection of ADH, the protein strongly connected with a low level of oxygen in all organisms, including plants, and known as a hypoxia marker. Additionally, we pointed out how the localization of the ADH enzyme was distributed within the tissue of roots. Finally, we wanted to determine how priming (pre-treatment) influences long-term memory and stress tolerance acquisition in WL-T and WL-S tomatoes. The function of these up-regulated proteins in terms of their possible role under low oxygen exposure is discussed.

## 2. Materials and Methods

### 2.1. Plant Material, Cultivation and Stress Treatment

**Figure 1:**
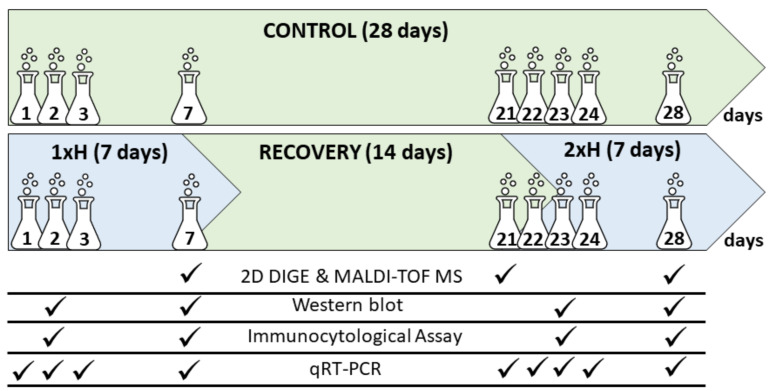
Scheme describing the experiment and time-points of sample collection for all analysis. The control plants of POL 7/15 and PZ 215 tomato accessions were grown under optimal conditions for 28 days (Ctrl), and samples were collected after 1, 2, 3, 7, 21, 22, 23, 24, and 28 days (marked with flasks). The POL 7/15 and PZ 215 accessions were exposed to hypoxia stress for 7 days by waterlogging (1xH). Some plants were devoted to collection of samples after 1, 2, 3 and 7 days (marked with flasks). The rest of the plants were maintained under optimal conditions for 14 days (Rec). Randomly selected plants were devoted to samples collected after 21 days. The rest of the plants were again exposed to hypoxia stress for 7 days (2xH), and samples were collected after 22, 23, 24, and 28 days.

Two tomato accessions, i.e., POL 7/15 (WL-T) and PZ 215 (WL-S), provided by KHiNO Polan (Krakow, Poland) and PlantiCo Zielonki Sp. z o.o. (Stare Babice, Poland), respectively, were characterized by contrasting responses to oxygen deprivation in the soil [17]. Seeds were sown in 40-cell multi-pots filled with Klasmann KTS-2 peat substrate (Klasmann, Geeste, Germany) containing 250–500 mg dm^−3^ N, 170–230 mg dm^−3^ P_2_O_5_, 320–500 mg dm^−3^ K_2_O, and 80–120 mg dm^−3^ Mg. The plants were cultivated under controlled conditions in a greenhouse and were lit with High-Pressure Sodium HPS lamps to sustain a 16/8 h light/dark regime at 26 °C during the day and 24 °C during the night. Minimum photosynthetic photon flux density (PPFD) on plant level during the day was 80 ± 20 µmol m^−2^ s^−1^. Plants were fertilized with growth fertilizer 3 days before the first waterlogging and every 3 days from the end of the first waterlogging stress with regenerative fertilizer. The growth fertilizer contained 7.5 g of Superba^TM^ Green Forte (8.2% N, 11.5% P_2_O_5_, 36.1% K_2_O, 2.8% MgO, 5.7% S, 0.23% Fe, 0.14% Mn, 0.03% Zn, 01% Cu, 0.04 B, 0.003% Mo) (Yara International ASA; Oslo, Norway), 7.0 g of YaraLiva CALCINIT Flakes (15.2% N, 27.5% CaO) (Yara International ASA; Oslo, Norway), and 3.0 g KRISTA^TM^ MAG (11% N, 15% MgO) (Yara International ASA; Oslo, Norway) diluted in 10 dm^3^ H_2_O, whereas the regenerative fertilizer consisted of 11.5 g, 8.8 g, and 4.4 g, respectively, of the same components as the growth fertilizer. The pH of the fertilizers was adjusted to 5.8 with nitric acid (V), and the final soil electrical conductivity (EC) was 2.3 ms cm^−1^. After 21 days of cultivation, plants were divided into four groups: (1) untreated plants, cultivated under optimal conditions (Ctrl), (2) non-primed plants, waterlogged for 7 days only once (1xH), (3) plants after 7 days of waterlogging and 14 days of recovery (Rec), and (4) primed plants waterlogged for 7 days and after 14 days of recovery, then waterlogged again (2xH) (Figure 1). The experiment consisted of four multi-well pots in a randomized block design. There were 40 plants per multi-well pot and 160 plants per group (a total of 640 plants per experiment).

The plants from the control group (Ctrl) stayed unstressed throughout the experiment and were watered as needed to ensure optimal growth conditions. The root zone and hypocotyls at a height of around 5–8 cm of the plants in the 1xH group were waterlogged for 7 days in deep plastic trays (600 × 400 × 200 cm). Then, plants were taken out of the water and stayed unstressed for 14 days (Rec). Next, plants from the 1xH group were waterlogged for the second time for another 7 days (2xH). The morphological diversity in the response of both tomato accessions after the first (1xH) and second (2xH) exposition to hypoxia stress was presented in Supplementary Figure S1. Oxygen levels in the water and in the air were periodically monitored using a dissolved oxygen (DO) meter (HI 2040-02 edge, Hanna instruments; Woonsocket, RI, USA). During waterlogging, the oxygen level in the water reached 2.1 mg dm^−3^, which confirmed hypoxic conditions [20], whereas in the air, the dissolved O_2_ level was 9.0 mg dm^−3^.

### 2.2. Soluble Protein Extraction

The total protein extracts were obtained from roots of two tomato accessions using the method of Bohler et al. [21] with minor modifications. For 2D DIGE, the roots were collected from Ctrl, 1xH, Rec and 2xH plants at 7 d, 21 d and 28 d of the experiment (Figure 1). For elimination of risk of confounding proteins associated with plant growth and development with proteins associated with waterlogging tolerance, control probes were collected at each time point (7 d, 21 d, 28 d of experiment) and were pooled into 3 biological replicates. For stressed plant groups (1xH, Rec and 2xH), 3 biological replicates were prepared. Single replicates contained the roots from 5 independent plants. For Western blot, the roots were collected from Ctrl, 1xH, Rec and 2xH plants at 2 d, 7 d, 23 d and 28 d of the experiment (Figure 1). For each group (Ctrl, 1xH, Rec and 2xH), three biological replicates were pooled from 5 plants.

One hundred milligrams of roots were ground in liquid nitrogen. The obtained powder was suspended in extraction buffer (50 mM Tris pH 8.0, 25 mM EDTA, 0.5 M thiourea, 0.5% DTT) and centrifuged in 4 °C at 15,000 rcf for 15 min. To precipitate proteins, 300 µL of the supernatant were mixed with 1.5 mL of the precipitation solution (20% trichloroacetic acid, 0.1% DTT in ice-cold acetone), incubated for 1 h in −20 °C and centrifuged. The obtained pellets were washed with iced (−20 °C) acetone containing 0.1% DTT and centrifuged. The washing step was repeated three times and then the protein pellets were dried under vacuum. The dried pellets were resuspended in 150 µL of 4 M urea, 4% CHAPS, 30 mM Tris, pH 8.0 and incubated for 16 h at 4 °C. Next the samples were mixed by pipetting and centrifuged for 3 min. The supernatant was transferred to a new tube and stored at −80 °C. Protein concentration was determined using the Pierce BCA Protein Assay Kit (Thermo Fisher Scientific, Waltham, MA, USA) and RC DC Protein Assay Kit I (Bio-Rad, Hercules, CA, USA) according to the manufacturers’ instructions. 

### 2.3. D DIGE, Quantitative Protein Analysis, MALDI-TOF MS Analysis

A 2D DIGE was undertaken to compare protein abundances between treatments, i.e., 1xH and 2xH after 7 days of waterlogging and at day 14 of the recovery period (Rec) in accordance with Ctrl for both tomato accessions. Prior to electrophoresis, all protein extracts and a pooled internal standard were labeled with DIGE fluorescent dyes (GE Healthcare, Uppsala, Sweden). A total of 30 µg of protein samples were labelled with Cy3 or Cy5 dyes, at a ratio of 4 pmol μg^−1^ protein. A pooled protein sample containing equal amounts of all samples in the experiment was labeled with Cy2 using the same protocol. The labelling reactions were carried out in the dark and on ice for 30 min. Next, 1.0 μL of 10 mM lysine was added to each sample to stop the reaction and after that, the samples were vortexed, centrifuged and incubated in the dark for 10 min on ice. Next, the Cy2, Cy3, and Cy5 labeled samples were mixed and the volume was adjusted to 450 μL by adding the lysis buffer (7M urea, 2 M thiourea, 0.5% CHAPS and bromophenol blue), followed by the addition of 9 μL of ampholytes and 2.7 μL of DeStreak rehydration solution (GE Healthcare, Uppsala, Sweden) for the 24 cm gradient (pH 4–7) ReadyStrip™ IPG strip (BioRad, Hemel Hempstead, UK). Prior to the cup loading, the ReadyStrip™ IPG strips were rehydrated overnight with 450 µL of rehydration solution (3 mL of DeStreak solution (GE Healthcare, Uppsala, Sweden) and 15 µL of ampholytes). Isoelectric focusing (IEF) was carried out on an Ettan IPGphor Manifold (GE Healthcare, Uppsala, Sweden) in an IPGphor unit (GE-Healthcare) with the following protocol: 300 V for 2 h, gradient to 1000 V over 2 h, 1000 V for 2 h, gradient to 2000 V over 2 h, 2000 V for 2 h, gradient to 4000 V over 4 h, 4000 V for 2 h, gradient to 8000 V over 4 h, 8000 V, until ~120,000 V h were reached at 20 °C, with a maximum current setting of 50 μA per strip. After equilibration, reduction and alkylation of cysteines using 10 mg DTT and 250 mg iodoacetamide (IAA), respectively, the strips were placed on 12.5% acrylamide precasted gels (Serva Electrophoresis, Heidelberg, Germany) and followed by sealing with 0.5% agarose solution for second-dimension electrophoresis. The SDS gels were electrophoresed at 17 W/gel at 20 °C until the blue leaves the gel. DIGE labeled gels were scanned in Typhoon FLA 9500 (GE Healthcare, Uppsala, Sweden) and were analyzed using ImageQuantTL software ver.8.1 (GE Healthcare, Uppsala, Sweden), and then were subjected to the differential expression of the proteins using SameSpots software ver. 5.0.1.0 (TotalLab, Newcastle upon Tyne, UK). A total of 12 gels were run comprising protein samples from 8 different sets of treatments and 3 biological replications each (Supplementary Table S2). Alignment and normalization with the internal standard allow comparison of the spots between repetitions because the same internal standard was run on each gel. Fold change for the comparison of the spot intensities between different treatments was calculated based on the ratio of the normalized, log-transformed spot intensities. Statistical significance of the relative change of accumulation of protein spots was determined using Student’s *t-*test, and the full list of identified spots in all the replicates gels was presented as Supplementary Data S1, and spots selected for picking were presented in the 2D-DIGE map (Supplementary Figure S2). For cluster analysis of protein abundance values, the web-based software NIA array analysis tool [22] (http://lgsun.grc.nia.nih.gov/anova; accessed on 21 November 2021) was used, which allowed us to select statistically valid protein spots based on analysis of variance (ANOVA). Data were analyzed using the following settings: error model ‘max (average, actual)’, 0.01 proportion of highest variance values to be removed before variance averaging, 10° of freedom for the Bayesian error model, 0.05 FDR threshold, and zero permutations. A multivariate analysis was carried out in two steps: (1) hierarchical clustering was performed to check the entire dataset, and the results were represented in dendrograms using the cluster function of the software; (2) the entire dataset was analyzed by PCA. The settings used for the PCA analysis were co-variance matrix type, three principal components, one fold change and 0.4 correlation threshold for clusters. 

A total of 376 protein spots presenting differential accumulation were picked up from the gels, with the Ettan Spot Picker (GE Healthcare, Uppsala, Sweden) and trypsinized using an EVO2 workstation (Tecan Trading AG, Männedorf, Switzerland). The samples were solubilized in 0.7 μL of α-cyano-4-hydroxycinnamate (CHCA) solution (7 mg dm^−3^ in 50% acetonitrile (ACN) and 0.1% trifluoroacetic acid (TFA)) and spotted onto a MALDI plate. MALDI TOF/TOF analysis was performed with a TOF/TOF™ 5800 (AB SCIEX, Redwood City, CA, USA) mass spectrometer in MS and MS/MS mode. The 10 most intense peaks for each spot of the MS spectrum were selected for MS/MS acquisition. Database searching was carried out over on an in-house Mascot server version 2.6.1 (Matrix Science Ltd., London, UK) through Protein Pilot v4.5 (SCIEX, Redwood City, CA, USA) for database-dependent identifications. A first search was performed against the JGI-Tomato database (available online: https://phytozome.jgi.doe.gov, accessed on 20 November 2021) ver. ITAG2.4 (34,725 sequences; 11,955,943 residues) and a second one against the NCBI non-redundant protein sequence database (NCBInr) limited to the taxonomy *S. lycopersicum* (taxID 4107; 43,550 sequences; 19,903,915 residues). The mass tolerances were 100 ppm for precursor ions and 0.5 Da for fragment ions. Trypsin was used as enzyme allowing two missed cleavages. The variable modifications allowed were tryptophan oxidation and double oxidation, methionine oxidation and tryptophan to Kynurenin (is an artifact often observed during automatic digestion in the laboratory). Carbamidomethyl cysteine was set as fixed modification. When at least two peptides passed the MASCOT-calculated 0.05 threshold score of 58 and individual ions score >32, proteins were considered as identified. Additionally, if a high-quality spectrum was not matched to a protein, the interpretation was done manually and search parameters adjusted (semitryptic, single amino acid change, and post-translational modification) to increase the sequence coverage of identified proteins (Supplementary Data S2). The cellular component, biological process and molecular function of the identified proteins was assigned with Gene Ontology Annotation (GO; www.geneontology.org, accessed on 30 November 2021) using Blast2GO software ver. 2.3.5 (www.blast2go.de, accessed on 30 November 2021). For proteomic analysis, differentially abundant spots for each tomato accessions and all treatments were selected when difference between hypoxia-treated (1xH, 2xH and Rec) versus untreated plants (Ctrl) was statistically significant according to Student’s t-tests with *p*  <  0.01.

### 2.4. Validation of Some Key Candidate Proteins by Western-Blot

Western blotting was performed to compare protein abundances between treatments, i.e., Ctrl, 1xH and 2xH after 2 and 7 days of waterlogging for both tomato accessions. Tris-tricine SDS-PAGE was carried out according to Shägger and von Jagow [23] using the V10-WCDC unit (Scie-Plas, Cambridge, UK). Protein samples were prepared by mixing the protein preparation (p. 2.2.) containing 6 µg of proteins, with one volume of the Laemmli sample buffer (2×) (Sigma Aldrich, St. Louis, MO, USA) and incubating at 95 °C for 5 min. Along with protein samples, 1 µL of the PageRuler Prestained Protein Ladder Plus (Thermo Fisher Scientific, Waltham, WA, USA) was loaded onto the polyacrylamide gel. Electrophoresis was performed for 4.5 h at 200 V, 120 mA (two gels or 60 mA—one gel) at 4 °C. After electrophoresis, proteins from the gel were electroblotted onto a nitrocellulose membrane (pore size 0.2 µm) with the Trans-Blot SD Semi-Dry Transfer Cell (Bio-Rad, Hercules, CA, USA) using a transfer buffer containing 48 mM Tris pH 9.2, 39 mM glycine, 20% methanol, and 1.3 mM SDS. The transfer was performed at 10 V (limiting parameter), 400 mA at room temperature for 30 min. The membranes were stored between sheets of Whatman filter paper at room temperature. Prior to Western blotting, the effectiveness of the protein transfer was evaluated by staining the membrane with 0.1% solution of Ponceau S in 5% acetic acid. Stained membranes were digitalized using the Epson Perfection V750 Pro scanner. The membranes were subsequently destained by washing in 10 mM NaOH. Then, NaOH was removed by washing the membranes three times with MilliQ water and once with TBST buffer (10 mM Tris pH 7.5, 150 mM NaCl, 0.05% Tween 20). Each washing step lasted 2 min. The membranes were blocked at room temperature for 2 h in TBST buffer containing 3% skim milk (prepared from milk powder) and then probed with a primary antibody. Two rabbit polyclonal antibodies were used – one against the alcohol dehydrogenase (ADH, cat. no. AS10 685) and the other against actin (ACT, cat. no. AS13 2640), both provided by Agrisera (Vännäs, Sweden). The third primary antibody had specificity against the glyceraldehyde-3-phosphate dehydrogenase (GAPDH). It was provided by Abmart (Shanghai, China) in the form of three mouse monoclonal antibodies (cat. no. K4BYG6) corresponding to three N-terminal epitopes. All primary antibodies were used at 1:2,500 dilution in TBST buffer containing 1% skim milk. After washing in TBST (3 times for 10 min.), the blot was incubated for 1.5 h with a secondary antibody—either the anti-rabbit IgG or anti-mouse IgG antibody, both from goat and conjugated to alkaline phosphatase (Sigma-Aldrich, St. Louis, MO, USA). The secondary antibodies were applied at a 1:5000 dilution in TBST buffer containing 1% skim milk. After washing with buffers TBST (3 times for 10 min.), TBS (10 min.) and AP (100 mM Tris pH 9.5, 100 mM NaCl, 5 mM MgCl_2_, 3 times for 2 min.), the solution of BCIP and NBT was applied to detect the antigen–antibody complexes. This solution was prepared by mixing 3.3 µL of stock solutions (100 mg/mL NBT, 50 mg/mL BCIP, both in 70% dimethylformamide) per 1 mL of the AP buffer. After detection, the membranes were scanned (Epson Perfection V750 Pro scanner) and the resulting images were subjected to densitometry analysis using the ImageJ 1.51j8 [24] software (National Institutes of Health, Wayne Rasband, Bethesda, Maryland, USA). Integrated optical density values measured for each protein signal were normalized by the integrated optical density value of the 60 kDa Ponceau S band from the same lane. After normalization, average values and standard errors were calculated within biological groups (plants subjected to different treatments) and plotted onto column charts.

### 2.5. Expression of adh Gene by qRT-PCR Assay

For the qRT-PCR assay, the roots were collected at 1, 2, 3, and 7 days from the Ctrl, 1xH, and 2xH groups and also after 14 days of Recovery (Rec). Roots were frozen immediately in liquid nitrogen after collecting and then stored at −80 °C until RNA extraction. Total RNA isolation was performed with Direct-zol RNA MiniPrep Plus (Zymo Research, Irvine, CA, USA) according to the manufacturer’s instruction. RNA extracts were treated with 1 U µL^−1^ RNase-free Dnase I (Thermo Fisher Scientific, Waltham, MA, USA) and 40 U µL^−1^ of RiboLock RNase Inhibitor (Thermo Fisher Scientific, Waltham, USA) to prevent DNA contamination. RNA quality and quantity were monitored by gel electrophoresis under denaturing conditions. The qRT-PCR assay was conducted for *alcohol dehydrogenase* 2 (*adh2*) gene (GenBank Acc. No. NM_001247170), for which a pair of primers was designed, i.e., *adh-F* (5’-CACTGCCTCAACTGAGTAAAC-3’) and *adh*-R (5’-CAGCAGCTTTGCAACGAA-3’), generating a fragment of 128 bp. Both sequences were submitted to BLAST of the National Center for Biotechnology Information to verify their analytical specificity, whereas the formation of dimers, hairpins and melting temperature was assessed with OligoAnalyzer 3.1 software (https://eu.idtdna.com/pages/tools/oligoanalyzer, accessed on 30 November 2021). Transcript levels were normalized to the expression level of *ef1* gene (GenBank Acc. No. X53043.1) using the primers *ef1*-F (5′- TACTGGTGGTTTTGAAGCTG-3′) and *ef1*-R (5′-AACTTCCTTCACGATTTCATCATA-3′), generating a fragment of 175 bp. qRT-PCR and relative quantification of *adh2* gene expression were conducted according to the method described by Kęska et al. [25].

### 2.6. Histological and Immunocytological Analyses

For histological analysis, samples of roots from 28-day-old control plants were collected and fixed overnight at 4 °C in a solution of in 5% (*w*/*v*) glutaraldehyde (GLA) in 0.1 M phosphate-buffered saline (PBS) (pH 7.2). Next, the samples were washed four times in phosphate buffer and dehydrated gradually in an ethanol series, from 10 to 100 % (*v*/*v*). The fixed tissues were embedded in Technovit 7100 synthetic resin and 5 μm-thick sections were cut, stained with 0.1 % (*w*/*v*) toluidine blue (TBO), and finally mounted in Entellan (Merck, Darmstadt, Germany) according to the procedure described by Popielarska et al. [26]. Observations and documentation were performed with bright-field illumination using a Nikon Eclipse E400 microscope equipped with a Zeiss AxioCam MRe digital camera and Zeiss AxioVision 3.0 software (Carl Zeiss Microscopy, White Plains, NY, USA) and a Nikon DS-Fi2 with NIS-Elements 4.0 software.(Nikon Instruments Inc., Melville, NY, USA).

For immunocytological analysis, the parts of roots and leaf blades of two tomato accessions were fixed in 4% formaldehyde and 0.25% glutaraldehyde (GLA) in piperazine buffer 4 °C overnight then embedded in Steedman’s Wax and sectioned at 10 µm. The ADH was detected in plant material using rabbit primary antibody provided by Agrisera (AS10 685, Vännäs, Sweden), diluted 1:250 in 1% BSA in 1× PBS, pH 7.2 and with secondary goat anti-rabbit antibody conjugated with DyLight® 550 (Agrisera, AS111782) diluted 1:500 in PBS buffer for 2 h at 37 °C. To verify specificity of immunolabeling, a set of control experiments was performed, i.e., without the primary, secondary, or both antibodies (to confront with an autofluorescent signal for example of xylem cells walls). The chromatin of the nuclei was visualized with 7 ng mL^−1^ 4′,6′-diamidino–2-phenylindole dihydrochloride (DAPI, Sigma-Aldrich, St. Louis, MO, USA) in PBS. Sections were cover-slipped using Mowiol medium and viewed in an epifluorescence microscope Leica DM6000 B supported by LAS AF software [27] (Leica Microsystems GmbH, Wetzlar, Germany). All images of roots and leaf blades are taken as combination of fluorescence and Nomarski contrast (DIC).

## 3. Results

### 3.1. Waterlogging-Induced Changes in the Tomato Root Proteomes

Two-dimensional electrophoresis (2-DE) maps of roots representing four groups of treatments, i.e., Ctrl, 1xH, Rec, 2xH of PZ 215 and POL 7/15 accessions, were constructed to identify protein changes in the unstressed plants, in non-primed plants (waterlogged for 7 days only once), in plants after 14 days of recovery, and in primed plants (waterlogged for 7 days two times). A highly reproducible protein profile among replicates from the same accessions/treatment was detected for 376 protein spots, and they were picked from the gels (Supplementary Figure S2, Supplementary Data S1). 

**Figure 2:**
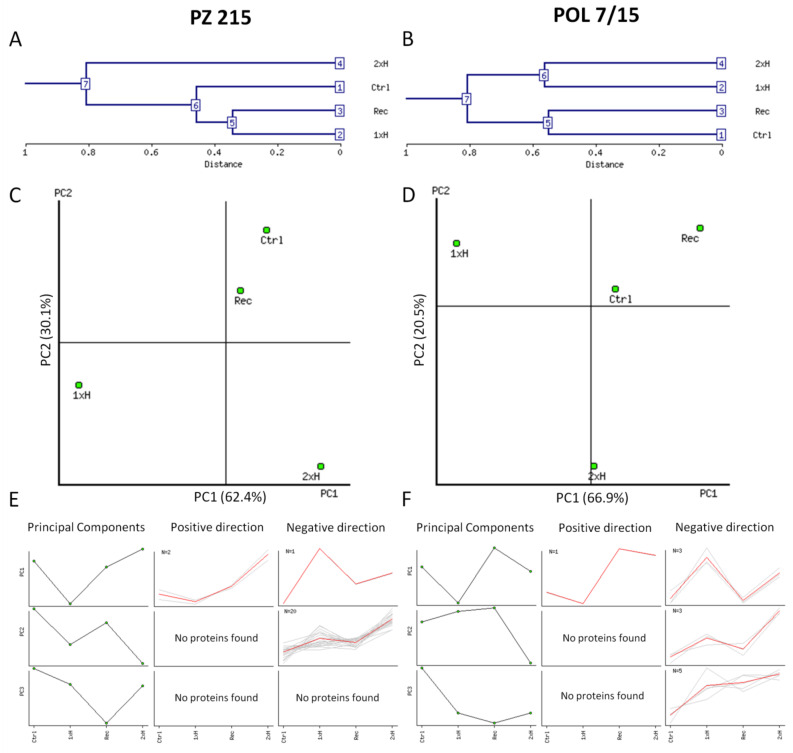
Statistical protein abundance cluster analysis using the ANOVA-based NIA array analysis tool of two tomato accessions, i.e., PZ 215 (**A**,**C**,**E**) and POL 7/15 (**B**,**D**,**F**), after first waterlogging, non-primed plants (1xH), after a 14-day recovery period (Rec), after secondary waterlogging exposure, primed plants (2xH) and untreated, control plants (Ctrl). (**A**,**B**)—Dendrogram showing hierarchical clustering of experimental conditions. (**C**,**D**)—Two-dimensional plots showing separation of samples plotted in the first and the second component space by Principal Component Analysis (PCA) and a short distance between samples in the component space is indicative of similarity in abundance profiles. (**E**,**F**)—Protein spot abundance clustering based on PCA. For each PC, two clusters of proteins were identified that were positively and negatively correlated with the PC. The degree of protein abundance change within a specific PC was measured by the slope of regression of log-transformed protein abundance versus the corresponding eigenvector multiplied by the range of values within the eigenvector. If the degree of protein abundance change exceeded the onefold change threshold, the protein spot was considered to be associated with the PC. Protein clustering was performed sequentially starting from the first PC. Proteins that were already clustered with a PC were not included in the clusters associated with subsequent PCs. Protein spots identified in this analysis (23 spots in PZ 215 and 12 in POL 7/15) are recorded in Supplementary Table S3.

Abundance data for all differential protein spots were analyzed using the NIA array analysis tool for hierarchical clustering of biological experiments and their repetitions (separately for each tomato accession) (Figure 2). We found that the experimental conditions could be divided into two large clusters in a dendrogram for both tomatoes; however, for PZ 215, cluster 6 (Ctrl, Rec and 1xH) and cluster 7 consisted of only 2xH (Figure 2A), while for POL 7/15, this was cluster 5 (Ctrl, Rec) and cluster 6 (1xH and 2xH) (Figure 2B). This clustering indicated that PZ 215 plants had protein abundance profiles different from those shown by POL 7/15 plants after waterlogging priming (2xH). The hierarchical clustering of biological repetitions confirmed that the data were reproducible for the experiments (Figure 2C–F). PCA results showed that in PZ 215, PC1 and PC2 explained 63.40% and 30.09%, and in POL 7/15, 66.95% and 20.55% of total variance, respectively (Figure 2C,D). PCA provided information on the relevance of each protein related to the discrimination of both tomato accessions and treatments. The 23 protein spots identified in PZ 215 and 12 spots in POL 7/15 were shown to be correlated with principal components and to be representative for distinct abundance clusters (Figure 2E,F). Correlationships between PCs and the different quantitative variable spots are indicated in the Supplementary Table S3.

All of the differential protein spots were subjected to MALDI TOF/TOF MS analysis and out of the 376 protein spots analyzed, 257 proteins were successfully identified with the MASCOT search engine with high confidence (Supplementary Data S2). A total of 209 and 172 proteins were identified in PZ 215 and POL 7/15, respectively. Out of the total number of identified proteins, 37 were commonly identified in both tomato accessions. When in the spot more than one protein was identified, it could be attributed to the presence of different isoforms or post-translational modification (Supplementary Data S2). 

**Figure 3:**
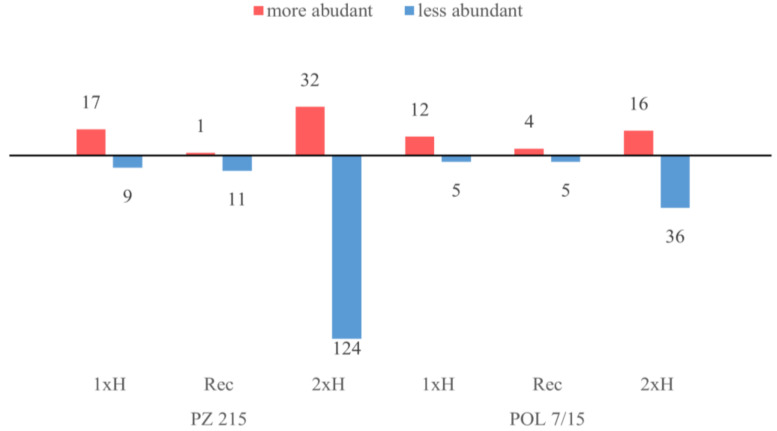
The number of more abundant (red boxes) and less abundant (blue boxes) proteins after an initial waterlogging (1xH), after a 14-day recovery period (Rec) and after secondary waterlogging exposure (2xH) in relation to the control group (Ctrl) in two tomato accessions, i.e., PZ 215 WL-S and POL 7/15 WL-T (*p*  <  0.01).

The differences in protein abundance (fold change) for PZ 215 and POL 7/15 were based on the ratio of 1xH/Ctrl, Rec/Ctrl and 2xH/Ctrl. We observed different mechanisms in response to waterlogging after the first and second exposure and in the recovery phase. Among the proteins that showed a significant change in abundance (*p* < 0.01), we found 26 and 17 responsive proteins under the first waterlogging (1xH) in PZ 215 and POL 7/15, respectively (Figure 3). In primed plants (2xH), we observed an increase in the number of proteins up to 156 in PZ 215 and 52 in POL 7/15, and most of these proteins showed significantly less abundance compared to the control group (Figure 3). Moreover, we found 12 (11 less abundant) and 9 (5 less abundant) proteins responsive for recovery after stress in PZ 215 and POL 7/15, respectively.

**Figure 4:**
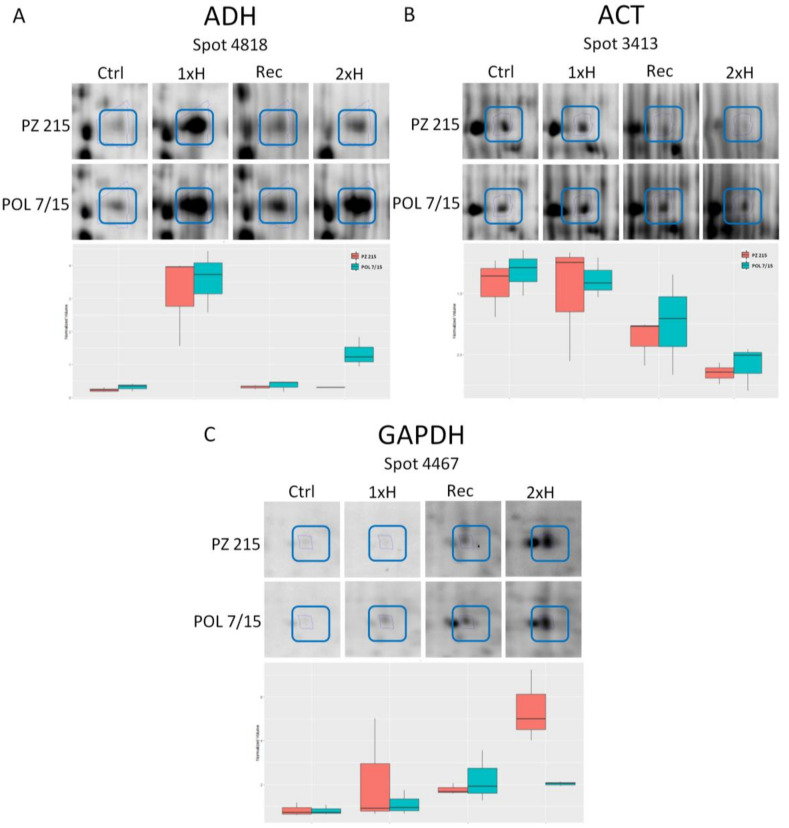
Protein spot images and abundance profiles of PZ 215 and POL 7/15 tomato accessions. Three protein spots were selected to illustrate differential expression profiles: (**A**)—spot 4818 (alcohol dehydrogenase, ADH), (**B**)—spot 3413 (actin, ACT), and (**C**)—spot 4467 (glyceraldehyde 3-phosphate dehydrogenase, GAPDH). Close-up regions of the 2D gels are shown (the respective differential proteins spots are marked by a blue box and from the left to right: unstressed plants (Ctrl), non-primed plants (1xH), plants after recovery (Rec), and primed plants (2xH). At the bottom, graphs of mean abundance values for the respective protein spots are shown (standard deviations were calculated from the means of the three biological repetitions).

Among the total identified protein spots showing significant differences, we found differences in constitutive patterns of proteome between two tomato accessions (Table 1). Within this category, we can differentiate three groups: 

(a) constitutively present proteins in PZ 215 that were more abundant in relation to the control, such as proteins of the cell wall macromolecule catabolic process (endochitinase, spot 3746), the sucrose biosynthetic process (fructokinase 2, spot 2065), the response to stress (germin, spot 2215), protein binding (heat shock protein, spot 4117; wound/stress protein, spot 4526), the defense response (pathogenesis-related protein-1A1, spot 4477), nitrate assimilation (glutamine synthetase, spot 1261), and also down-regulated proteins involved in proteolysis (cysteine proteinase cathepsin F, spot 3768 and subtilisin-like protease, spot 4667), the S-adenosylmethionine biosynthetic process (S-adenosylmethionine synthase, spot 3404), cell redox homeostasis (redoxin domain protein, spot 4272), and oxidoreductase activity (peroxidase 1, spot 1439); 

(b) constitutively present proteins in POL 7/15 that were more abundant, i.e., involved in the methionine biosynthetic process (5-methyltetrahydropteroyltriglutamate -homocysteine methyltransferase, spot 467), the glycolytic process (fructose-bisphosphate aldolase, spot 3473) and acohol dehydrogenase 2 (spot 145), two downregulated proteins connected with cell wall modification (pectinesterase, spot 4212), and nucleic acid binding (RNA binding protein-like protein, spot 4572);

(c) common proteins in both tomato accessions whose abundances increased, i.e., alcohol dehydrogenase 2 (spot 3439 and spot 4818) (Figure 4A), binding protein (osmotin-like protein, spot 4134), triosephosphate isomerase (spot 4043) involved in the glycolytic process, downregulated proteins involved in RNA binding (spot 3567 and spot 3378), the defense response (thaumatin-like proteinand peroxidase, spot 3418), and the oxidation–reduction process (peroxidase, spot 3418; UDP-D-glucose dehydrogenase, spot 3351).

In primed plants (after the second waterlogging exposure—2xH), we also found differences in constitutive patterns of proteome between two tomato accessions (Table 1). Moreover, we identified proteins whose abundances were significantly different in relation to the control and non-primed plants (after the first stress exposure—1xH). Within this category, we can differentiate six groups: 

(a) constitutively present proteins in PZ 215 that were more abundant, i.e., proteins of ATP binding (actin, spot 1796), protein binding (embryo-specific 3, spot 4804; wound/stress protein, spot 4406), alcohol dehydrogenase 2 (spot 3892), the cell wall macromolecule catabolic process (chitinase, spot 4768), the glycolytic process (enolase, spot 4804), glycine-rich RNA-binding protein (spot 4408), alpha-amylase inhibitor activity (Kunitz-type protease inhibitor, spot 4209), transmembrane transport (porin/voltage-dependent anion-selective channel protein, spot 2944), S-adenosylmethionine synthase (spot 1106), microtubule cytoskeleton organization (tubulin alpha-3 chain, spot 2511), and water-stress inducible protein 3 (spot 4482); 

(b) proteins which decreased in response to double waterlogging in PZ 215 involved in proteolisis (cathepsin B-like cysteine proteinase (spot 3529), leucyl aminopeptidase (spot 3270), the mitochondrial processing peptidase alpha subunit (spot 3305), subtilisin-like protease (spot 4702), protein disulfide isomerase (spot 3242), microtubule cytoskeleton organization (tubulin alpha, spot 1023 and 3337, and tubulin beta chain, spot 3664), the carbohydrate metabolic process (2 3-bisphosphoglycerate-independent phosphoglycerate mutase, spot 4722), ATP binding (actin, spot 3413 [Figure 4B]), the ATP biosynthetic process (ATP synthase, spot 3287 and 4766), the ATP metabolic process (V-type ATP synthase, spot 3321), protein folding (chaperone DnaK, spot 3212; chaperonin, spot 4705), the glycolytic process (fructose-bisphosphate aldolase, spot 3535; glyceraldehyde 3-phosphate dehydrogenase, spot 3452), and anaerobic respiration (succinate dehydrogenase flavoprotein subunit, spot 3208);

(c) only two constitutively present proteins in POL 7/15 that were more abundant, i.e., alcohol dehydrogenase 2 (spot 1849) and peptidyl-prolyl cis-trans isomerase (spot 4839); 

(d) proteins which decreased in response to double waterlogging in POL 7/15 involved in the oxidation–reduction process, among other things (methylmalonate-semialdehyde dehydrogenase, spot 908), peroxidase 57 (spot 3576), aerobic respiration (succinate dehydrogenase iron-sulfur protein, spot 4821);

(e) common proteins in both tomato accessions whose abundances increased, i.e., the glycolytic process (glyceraldehyde 3-phosphate dehydrogenase dehydrogenase, spot 2662 and spot 4467 (Figure 4C)), protein binding (LH2 PLAT domain-containing protein, spot 2602 and wound/stress protein, 4514), thiosulfate sulfurtransferase rhodanese domain protein (spot 4559);

(f) proteins which decreased in response to double waterlogging in POL 7/15 included microtubule cytoskeleton organization (tubulin beta chain, spot 959 and spot 3317), the oxidation-reduction process (cinnamyl alcohol dehydrogenase, spot 3392, UDP-glucose 6-dehydrogenase, spot 1063), the branched-chain amino acid biosynthetic process (dihydroxy-acid dehydratase, spot 4723; ketol-acid reductoisomerase, 3244).

After 14 days of recovery, we found protein spots that showed significant differences in constitutive patterns of proteome between the two tomato accessions (Table 1). In PZ 215, we identified only less-abundant proteins involved in the sucrose biosynthetic process (fructokinase 2, spot 1429), the carbohydrate metabolic process (2 3-bisphosphoglycerate-independent phosphoglycerate mutase, spot 3236), cysteine synthase (spot 3590), microtubule cytoskeleton organization (tubulin alpha-7-chain, spot 3337), and also in the pyruvate metabolic process (acetyltransferase component of pyruvate dehydrogenase complex, spot 3298). In POL 7/15, we found four protein spots whose abundances were significantly different in relation to the control, namely actin (spot 3825), osmotin-like protein (spot 4130), tubulin alpha-3 chain (spot 4260), and peptidyl-prolyl cis-trans isomerase (spot 4839). We identified three protein spots that decreased in relation to the control, namely peroxidases (spot 1213 and 4697) and pyruvate decarboxylase (spot 726).

### 3.2. Validation of Key Proteins by Western Blot

These analyses targeted three proteins for which their responsiveness to hypoxia stress was suggested by the proteomic data (see above). Immunoblotting was performed both for the POL 7/15 and PZ 215 plants which were subjected to hypoxia once (1xH), and twice (2xH) for 7 days with 14 days of regeneration between exposures. The root samples for analysis were collected after 2 days of the primary (1xH) and secondary hypoxia (2xH), as well as after 7 days of these exposures. In parallel, the root samples from the control (unexposed) plants were collected. 

**Figure 5:**
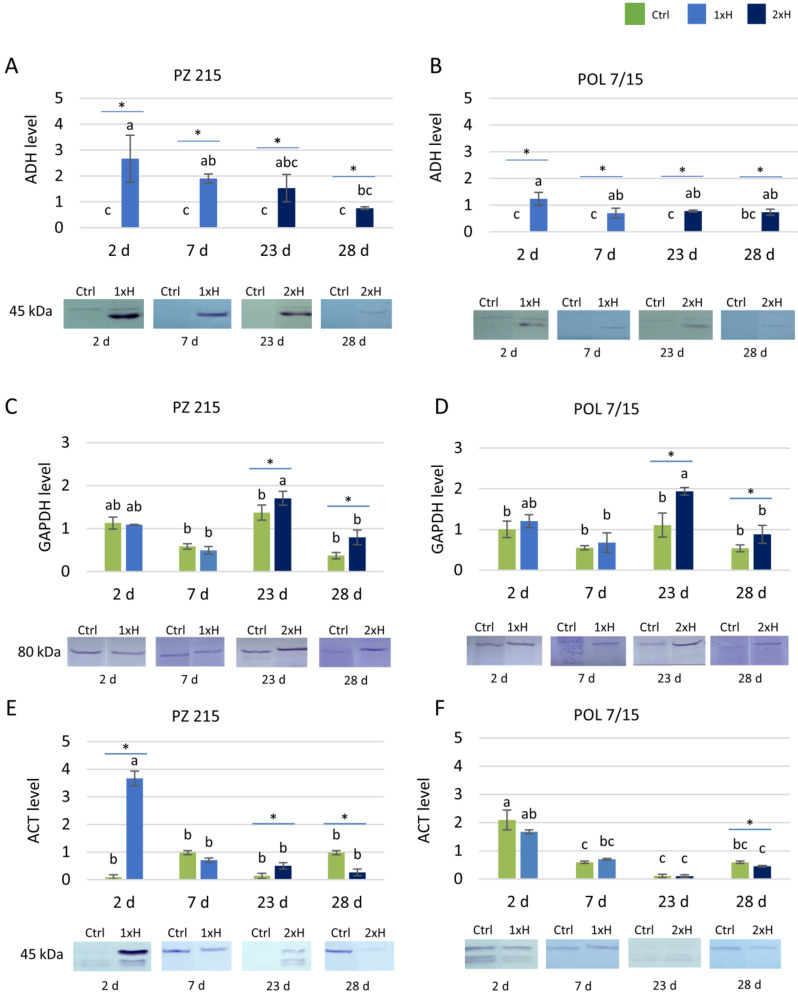
Western blot detection of three selected proteins that in 2-DIGE analysis showed significant differences between PZ 215 and POL 7/15 under waterlogging stress, i.e., alcohol dehydrogenase (ADH) (**A**,**B**), glyceraldehyde-3-phosphate dehydrogenase (GAPDH) (**C**,**D**), and actin (ACT) (**E**,**F**). Signal intensities obtained using densitometric analysis are shown in graphs; 2 d, 7 d and 23 d, 28 d—the 2nd and 7th day of the first (1xH) and second (2xH) waterlogging exposure, respectively. Data are expressed as the mean ± SD (standard deviation) of three independent biological replicates with *p* < 0.05 (Tukey’s post hoc test). Asterisks indicate a significant difference versus control plants. The same letters indicate no statistical differences between exposures and time-points (days).

Detection of alcohol dehydrogenase (ADH) revealed a 45 kDa protein which was present only in plants subjected to hypoxia (Figure 5A,B). Generally, ADH accumulation was higher for PZ 215 than for POL 7/15, with the only exception being the secondary hypoxia sampled after 7 days for which both accessions were comparable. Another observed regularity was the drop in the ADH level on the transition from the primary to secondary hypoxia. Moreover, the anti-ADH antibodies cross-reacted with a 50 kDa protein which was visible in the majority of samples collected after 2 days of hypoxia exposure (including controls). 

Antibodies against glyceraldehyde-3-phosphate dehydrogenase (GAPDH) detected a single protein of 80 kDa (Figure 5C,D). Hypoxia stress exposure-versus-control differences in GAPDH accumulation were evident mostly for POL 7/15, but also for PZ 215 which was waterlogged twice (2xH) showing greater accumulation.

Altogether, three proteins were detected with anti-actin (ACT) antibodies; the sizes of these proteins were estimated at 45, 40 and 37 kDa (Figure 5E,F). The two smaller proteins were observed in the majority of samples collected after 2 days of hypoxia (including controls), whilst they were not detected in the samples collected at the end of the exposure. For actin, two hypoxia-related effects were observed. In PZ 215, the plants waterlogged for 2 days (both 1xH and 2xH) showed an increase in ACT accumulation in comparison to the untreated plants (Ctrl); this was particularly evident in the case of primary hypoxia. Moreover, both accessions showed a decrease in actin accumulation in samples collected after 7 days of exposure.

### 3.3. Expression Profile of adh2 Gene 

**Figure 6:**
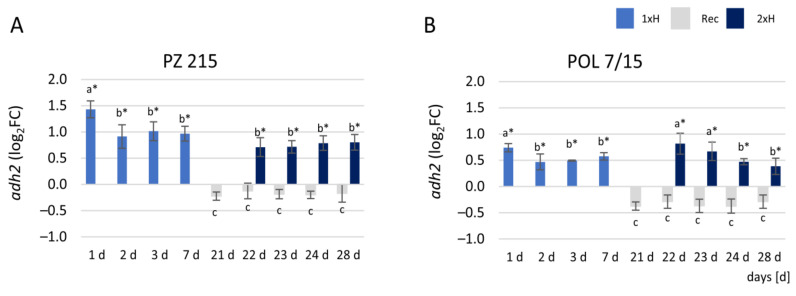
Expression profiles of the *adh2* gene in PZ 215 (**A**) and POL 7/15 (**B**) tomato accessions under waterlogging stress, respectively. Data are expressed as the mean ± SD (standard deviation) of three independent biological replicates and three technical replications with *p* < 0.05 (Tukey’s post hoc test). Asterisks indicate a significant difference versus control plants. The same letters indicate no statistical differences between exposures and time-points (days).

The expression level of the alcohol dehydrogenase (*adh2*) gene that is involved in the response to hypoxia was differentially regulated between the PZ 215 (WL-S) and POL 7/15 (WL-T) tomato accessions. The relative expression level of the *adh2* gene was approximately two-fold higher in PZ 215 plants under waterlogging stress than in POL 7/15 plants (Figure 6A,B). However, in PZ 215, *adh2* gene expression presented the highest level on day 1, then decreased and was up-regulated to the same level throughout the end of the experiment. In POL 7/15, the first waterlogging exposure enhanced the expression of the *adh2* gene just 1 day after stress induction, but the up-regulation decreased on day 2 and was at the same level up to day 7 of stress; however, after the second waterlogging (2xH), *adh2* gene expression increased from day 1 to 2 (22–23 days of the experiment), then decreased. In recovered plants (Rec), the *adh2* gene was down-regulated in sensitive and tolerant accessions (Figure 6A,B).

### 3.4. Root Sections and Immunohistochemical Localization of ADH in Root Tissues

**Figure 7:**
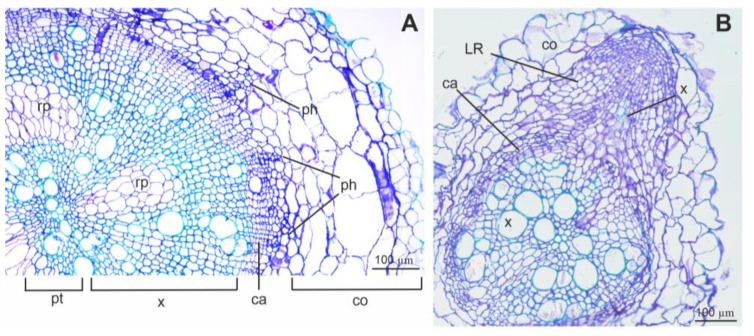
Technovit-embedded cross sections of taproot (**A**) and lateral root (**B**) of 28-day-old *Solanum lycopersicum* control plants. A Location of cortex and stele with vascular tissue including secondary xylem (*x*), and phloem (*ph*); visible circle of cambium (*ca*), ray parenchyma (*rp*) and the pith (*pt*) with remnants of primary xylem. B Induction of a second lateral root (*LR*) with xylem formation. Scale bar = 100 µm.

The observation of cross sections of roots from 28-day-old control plants with bright-field illumination revealed advanced secondary growth (Figure 7A) or its induction, especially in the lateral roots (Figure 7B). The circle of cambium was clearly visible, as well as the effects of its activity, which is the secondary xylem, ray parenchyma, and phloem (Figure 7A). Primary growth tissues (the pith with primary xylem and cortex) were also observed. The growth of the lateral roots started from the outer part of the stele (Figure 7B). 

**Figure 8:**
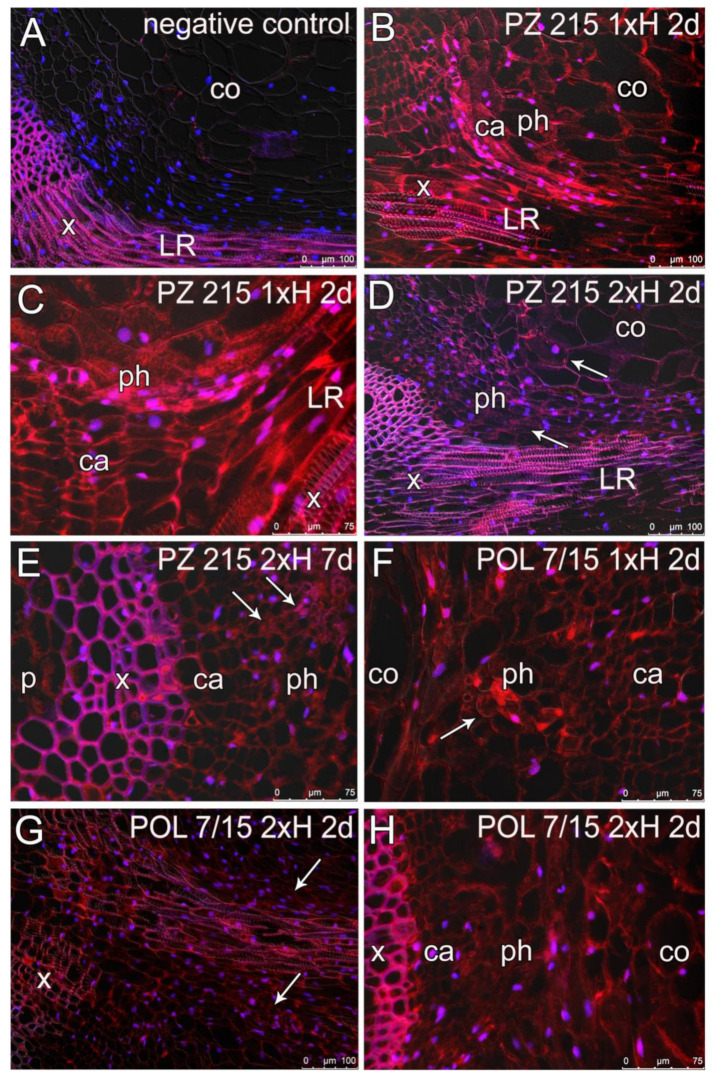
Steedman’s Wax-embedded cross sections of roots of *Solanum lycopersicum*. (**A**) Root tissue of control plants of POL 7/15 accession labelled omitting the primary antibody; lack of signal in all of the tissue (i.e., cortex cells, *co* apart from autofluorescent signal of xylem, *x* of lateral roots *LR*). (**B**–**E**) Sections of PZ 215 accession tap root and lateral roots after the first (1xH, (**B**,**C**)) and second (2xH, (**D**,**E**)) waterlogging. (**B**) Part of the stem of the taproot with lateral root (*LR*) formation. The ADH signal observed in vascular tissues: parenchymal cells of xylem (*x*), cambium (*ca*), phloem (*ph*) and surrounding cortex cells (*co*) of the taproot. (**C**) A magnification of the details from A. (**D**) Part of the stem of the taproot with lateral root (*LR*) formation; the ADH signal observed in vascular tissues: xylem (*x*), phloem (*ph*) and surrounding cortex cells (*co*) of taproot; signals mainly in phloem and cortex cells (*arrows*). (**E**) A magnification of the details of cambium and phloem. **F**–**H** Sections of POL 7/15 accession tap root after the first (1xH, F) and second (2xH, G, H) waterlogging. (**F**): Magnification of tissues with the ADH signal observed in vascular tissues: phloem (*ph*, *arrows*) and in parenchymal cells of xylem (*x*), cambium (*ca*), surrounding cortex cells (*co*) of the taproot. (**G**) Part of the stem of the taproot with lateral root (*LR*) formation; the ADH signal observed in vascular tissues: phloem (*ph*, *arrows*) and a near absence of signal in the surrounding cells of the taproot. (**H**) The ADH activity labelled mainly in phloem cells (*ph*) and cambium (*ca*) in taproot and cortex cells; the signal detected also in the parenchymal cells of the xylem (*x*). Scale bar = 100 µm (**A**,**B**,**D**,**G**), 75 µm (**C**,**E**,**F**,**H**).

For immunohistological analysis, the negative controls were performed, omitting the primary antibody (Figure 8A and Supplementary Figure S3C). No signal was detected in cortex cells, phloem or cambium, only the strong autofluorescence of the xylem cell walls was visible (Figure 8A). In the roots of control plants, the punctate signal of ADH was noted in cambium, phloem and cortex cells (Supplementary Figure S3A,B). In leaves chosen for negative control reaction and in samples from control plants, only the autofluorescent signal of the chloroplasts was detected (Supplementary Figure S3D,E). 

For both accessions after 2 and 7 days of two waterlogging stresses (1xH and 2xH), the signal of ADH detection was mainly observed in the cytoplasm of the cortex (parenchyma cells) and in vascular tissue (Figure 8 and Supplementary Figure S4). The signal of ADH activity was present after 2d of 1xH waterlogging for the PZ 215 accession in cells of vascular cambium/differentiating xylem, phloem, and in the cytoplasm of cortex cells. ADH activity was observed mostly in the stem of the taproot (TR) near lateral roots (LRs, Figure 8B and Supplementary Figure S4E). ADH detection was also noted in the epidermis and a few cells of parenchymal cells of pith (Supplementary Figure S4A,B,F). The signal of ADH was also observed in the cytoplasm of vascular tissue of LR (Figure 8B–E). For the POL 7/15 accession for 2 and 7 days after two waterlogging stresses (1xH and 2xH), the activity of ADH was observed in the same tissue as for the PZ 215 accession (Figure 8F–H). The aerenchyma formation was not detected in the cortex of the taproot or in lateral roots (Figure 8 and Supplementary Figure S4). 

## 4. Discussion

In the present work, we focused on roots, the first organs in which the defense against waterlogging stress is determined. Moreover, the physiological injuries in root tissue could be more severe than in shoots [9], which may cause permanent decreasing in crops yield [10]. For the first time, this study showed proteome profiles of sensitive and tolerant tomato accessions under two hypoxia exposures. The cluster method analysis of more abundant proteins versus a control group indicated similar protein profiles after the first and second hypoxia exposures for the WL-T tomato, and on the other hand, for the control and recovery period, which suggested that this accession responds along a similar pathway to the first and second stress factors. The reaction of the WL-S accession after the second hypoxia exposure was strikingly different because of the distinct protein profile in comparison with other exposures. Moreover, the protein profiles after the first hypoxia and recovery were similar.

### 4.1. Global Proteome Analysis after the First Waterlogging (1xH). Different Response of WL-S and WL-T Tomato Accessions

**Figure 9:**
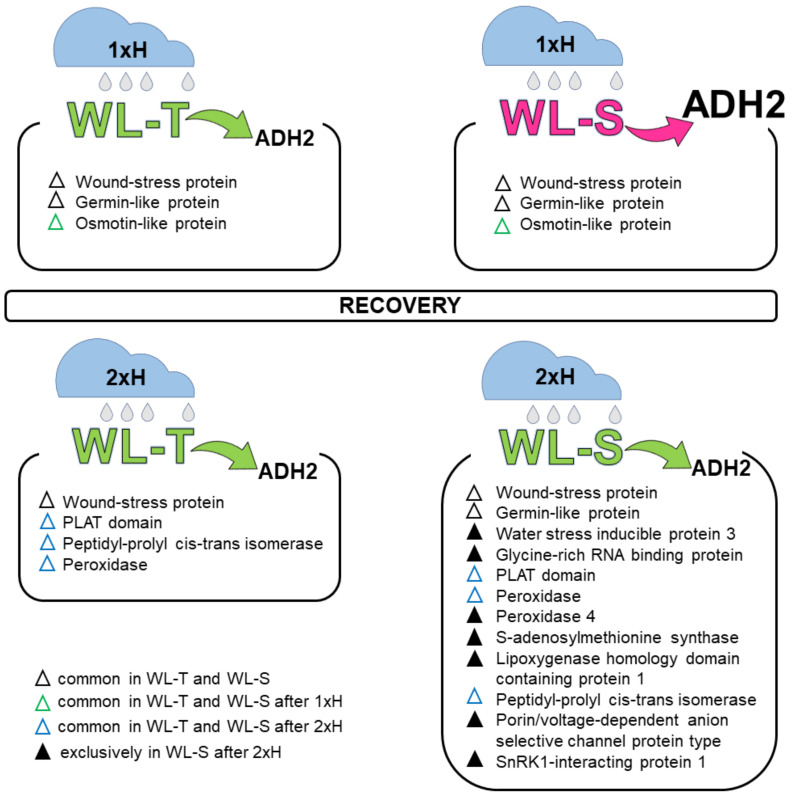
Model of protein expression in response to hypoxia stress in waterlogging-tolerant (WL-T) POL 7/15 and waterlogging-sensitive (WL-S) PZ 215 tomato accessions. After the first waterlogging (1xH), the up-regulation of DEGs proteins was the same for both of accessions, except ADH2, which was low for WL-T, and several-fold higher for WL-S. The expression of ADH2 after the second waterlogging (2xH) was similar for both accessions; however, the greater abundances of proteins were exclusively found in WL-S after 2xH, and these proteins could be candidates for hypoxia priming in tomatoes.

After the 1xH exposure, the protein profiles of WL-T and WL-S showed different responses. The common up-regulated proteins for both accessions were alcohol dehydrogenase (ADH), glyceraldehyde-3-phosphate dehydrogenase (GAPDH), triosephosphate isomerase (enzyme of glycolysis process) or germin-like and osmotin-like proteins (various biotic/abiotic stresses), among others. Some unique proteins for WL-T are 5-methyltetrahydropteroyltriglutamate-homocysteinmethyltransferase (participates in methionine metabolism), actin, nuclear RNA binding protein, and fructose-biphosphate aldolase (one of enzymes on glycolysis) [28] (Figure 9). Some unique proteins for WL-S are glutamine synthetase (nitrogen metabolism) [29], fructokinase-like (plastid protein), and heat shock protein 70 (biotic and abiotic stress response) [30]. Greater abundances at a similar level of fructose-biphosphate aldolase were described for tolerant and sensitive cultivars of the peanut [31]. The greater abundance of glutamine synthetase enhanced physiological tolerance under salinity, drought, and oxidative stress conditions in rice [29]. The greater abundance of heat shock protein 70 was noted for waterlogging-tolerant barley genotype [30], similarly as in the current study.

GLP proteins are specifically induced during germination; however, they can play diverse roles, including biotic and abiotic stress resistance [32]. The subgroups of GLPs have different enzyme functions that include the two hydrogen peroxide-generating enzymes, oxalate oxidase (OxO) and superoxide dismutase. Salinity and drought are the most often reported abiotic stresses for GLP induction. However, data concerning GLP expression under hypoxia or anoxia stress are scarce; as an example, we can mention the greater abundance of OXO-type GLPs in the response to hypoxia in wheat roots [33]. In the present research, a greater abundance of GLPs (Solyc01g102400) was detected for WL-T after the first exposure and for WL-S under the first and second hypoxia, which suggests that this protein could be involved in the adaptation to low oxygen conditions. 

Osmotin-like proteins (OLPs) are involved in the defense against abiotic and biotic stresses in plants, especially through the increase in the expression of genes coding ROS-eliminating enzymes. In the present study, a high expression amount of OLPs was detected for both of the accessions after the first waterlogging. Conversely, among the abiotic stresses connected with OLP abundance, drought, salinity, and low and high temperatures are noted [34].

The ADH, GAPDH and ACT protein amounts are discussed in Section 4.3. However, it is worth mentioning that parallel amount variations of the listed proteins with different masses were noted in the present research. Enzymes can be modified during the post-translational processes, leading to the creation of isoforms. GAPDH is identified as a glycoprotein, which requires glycosylation after translation [13]. It has been reported that under flooding, glycosylation and other post-translational protein modifications are down-regulated, especially in roots, rather than in shoots [35]. 

### 4.2. Global Proteome Analysis after 2xH. Candidates for Priming and Immune Tolerance Acquisition

The changes in the metabolic pathways relevant to the priming of organisms cause a distinct metabolic remodeling. Among all proteins identified and highly accumulated after the second waterlogging were proteins related to the abiotic stress response. In the present research, the responses of more and less abundant protein expression were the strongest in WL-S after 2xH. The high number of detected proteins could suggest that these molecules are essential for the adaptation to hypoxia stress and priming [8]. However, in comparison with other exposures, the highest number of some proteins was also less abundant in WL-S after 2xH. A similar phenomenon was reported for wild-type barley and transgenic plants with an overexpression of phytoglobin, the molecule which increases the survival rate of plants under hypoxia [8]. In the mentioned research, it was found that many proteins were less abundant in phytoglobin overexpressing barley, but not in the wild type, which was more susceptible to hypoxia. A similar relation was noted for the transcriptomic analysis of sensitive and tolerant plants, where the highest number of regulated transcripts was noted for sensitive cucumber after recurring hypoxia [25]. Data concerning the response of priming versus non-priming plants revealed the increasing number of differentially expressed genes for salinity priming rice [36] and cold priming *Arabidopsis* [37]. Generally, a higher reaction level of response (changes in hormones, metabolites, and other signals) in primed plants was reported [38].

The more abundant proteins—especially those detected in WL-S—could suggest that they are required for the acquisition of hypoxia tolerance (Figure 9). Another enzyme—apart from GAPDH mentioned above—involved in metabolic adjustment and important for glycolysis is enolase (or phosphopyruvate hydratase). The increase in enolase was reported for flooded tomatoes [14], soybeans [39], and peanuts [31]. These reports and the present study suggest that this glycolytic enzyme might help in intensification of glycolysis for generating energy under hypoxia stress. 

Ethylene biosynthesis begins with the conversion of methionine to S-adenosyl-methionine (SAM), catalyzed by SAM synthase. The mediating role of this simple two-carbon plant growth regulator in response to biotic and abiotic stresses has been noted [40]. In the present paper, the greater abundance (fold change 3.9) of one of the isomerases of SAM synthase was detected in WL-S after the second waterlogging. This agrees with results from wheat plants with a greater abundance of this enzyme after waterlogging priming [41].

SNRK1 is an evolutionary conserved central regulator of cellular metabolism related to energy metabolism under optimal growth and stress conditions [42,43]. This protein is considered to be an integrator of low energy signaling and acclimation towards different stress responses. In *Arabidopsis,* activation of SnRK1 was followed by reprogramming of transcription and an effect on many genes, which promotes long-term stress adaptation [44]. The pivotal role of SnRK1 in the regulation of carbohydrate metabolism under sugar starvation and energy crisis was well described under submergence and also under non-stress conditions, when tissue grows very actively [45].

RBPs are proteins that bind to RNA molecules, enabling their processing and metabolism, such as splicing, polyadenylation, editing, stabilization, localization, and translation. The start of different stresses caused the RNA and also RNA-related molecules to become engaged in the regulation of the response to the unfavorable conditions. The overexpression of glycine-rich RBPs was reported for *Arabidopsis* under abiotic stresses and enhanced the tolerance for cold, salinity and oxidative stress [20]. PRX4 belongs to the class III peroxidases (PRX), a plant-specific multigene family especially involved in the generation and detoxification of reactive oxygen species (ROS) [46]. ROS play a role as signaling molecules; however, they also cause protein, lipid, and nucleic acid oxidation, which causes cell necrosis [47]. PRX are one of the enzymes which reduce ROS under biotic and abiotic stresses and are considered an indispensable gene for crop improvement [48]. Anaerobic fermentation, which dramatically increases in waterlogging plants, involves the formation of a high amount of ROS. The increase in expression especially of the *PRX4* gene was observed in *Arabidopsis* during hypoxia [49]. In the present study, greater accumulation of PRX4 (Solyc04g071890, located on chromosome 4) was observed for WT-S after 2xH; however, the greater abundance of PRX (Solyc01g105070, located on chromosome 1) was noted for both accessions after the second waterlogging.

PPIases, also called immunophilins, are a family of ubiquitous proteins with multiple functionalities in plants [50]. Two subfamilies are known: cyclophilins (CYPs) and FK506 binding proteins (FKBPs). The basic role of cyclophilin is protein folding, which influences such phenomenon as mRNA processing, protein degradation, signal transduction, chaperon activity, and generally maintaining homeostasis in the cell [51]. It has been demonstrated that one of their tasks is adaptation to a broad range of abiotic stress conditions, as has been noted in *Arabidopsis* [52] and rice [53]. Hypoxia causes a reduction in pH of cytosol in plant cells. This is caused by the low concentration of ATP, which affects the low activity of the plasma membrane proton pump, and the accumulation of weak acids from anaerobic carbohydrate pathways [41]. One of the roles of PPIase is the stabilization of pH homeostasis during intracellular acidification, which has been demonstrated for over-expressing *Arabidopsis* in the presence of acetic acid [54]. Among the different stress conditions, such as drought, oxidative stress, salinity, or wounding [50], there is no information concerning the relationship of cyclophilins and hypoxia conditions. However, regarding the expansion of cyclophilins in plants, its role should be taken into consideration for hypoxia exposure.

Water-stress-inducible protein 3 is a member of the ASR (ABA/water stress/ripening) protein family involved in adaptation to drought stress which functions as a transcription factor [55]. The ASR gene family is a key component of several regulatory networks with functional duality in plants [56]. However, among different abiotic factors, salt and heat–cold stresses are reported to have a relationship with ASR [56]. On the other hand, abscisic acid (ABA) seems to play a crucial role in coordination of various stress signals, both during water deficit and waterlogging [57].

As was mentioned above, different forms of stress may activate and utilize the same proteins, such as PPIase, or signaling molecules, such as ABA. In the present study, another protein (fold change 3.7 for both accessions) involved in biotic and abiotic stress responses is wound/stress-related protein. The accumulation of this protein has been observed in tomato seedlings in response to salt stress and wounding [58]. The functional mechanism possibly involved ROS signaling, as was suggested in research on light-induced wound response in *Arabidopsis thaliana* [59].

All responses to the environment, both biotic as well as abiotic stress, must be passed through cell signaling pathways. LOX1 is an enzyme belonging to the 9-LOX family which is involved in the formation of oxylipins, signal molecules considered to be important in development and stress response in roots [60]. In the present study, the containing protein called PLAT domain or LH2 (Lipoxygenase homology), was strongly up-regulated for both tomato accessions, particularly for WL-S after 2xH (fold change 6.2). This domain is understood to mediate interaction between membrane lipids and proteins, and was reported as critical for cold, drought and salt stress tolerance in *Arabidopsis*, probably through the regulation of the ABA pathway [61]. Moreover, the authors indicated the correlation between the expression of PLAT and the activity of pericycle cells and increasing number of lateral root primordia.

VDAC are most abundant in the outer mitochondrial membrane and are also involved in programmed cell death as a response to biotic and abiotic stresses. An increase in VDAC was noted for flood stressed *Beta vulgaris* roots [62].

Alpha-tubulin is the major constituent in microtubules, which play a role in maintaining cell shape and act as traps for vesicle transport and chromosome segregation. Microtubule depolymerization and reorganization are considered essential for plant survival under abiotic stress [63]. Additionally, microtubules may play sensory and translatory functions during the influence of stress factors [64]. However, contrary to animals [65], the present study is the first report regarding tubulin or microtubule reaction after hypoxia exposure in plants.

Some of the more abundant proteins mentioned above (such as GAPDH and enolase) are involved in carbohydrate consumption, one of the phenomena proposed as crucial for providing tolerance to hypoxia. The other critical processes proposed for survival of oxygen deficiency are the avoidance of oxidative stress, effective signaling, and mobilization of proteins responsible for RNA processing [1]. Some proteins proposed in the present paper as priming candidates are considered to be crucial factors in the response to various stress factors. This is in line with the observations of other researchers, who have suggested that one type of abiotic stress priming could induce tolerance to different types of stress [11].

### 4.3. Validation of Selected Proteins

Waterlogging stress influences plant energy metabolism, especially regarding pathways involving ADH or GAPDH [1,31,66]. An accumulation ion of both of these proteins was observed in WL-T as well as WL-S tomatoes after hypoxia exposure. For detailed studies, gene and protein validation for ADH and GAPDH were conducted. Additionally, the fluctuation in proteome profiles necessitated a profound analysis of the ACT protein.

In the present study, for both accessions, an increase in ADH2 protein after the first and second exposure using proteome analysis was noted. However, the difference in expression for sensitive and tolerant tomatoes after the first and also second waterlogging was evident. Interestingly, the proteome shows a greater abundance of ADH2 protein dedicated for a locus present on chromosome 6 (Solyc06g059740), but a downregulation of the ADH III-class protein for a locus present on chromosome 9 (Solyc09g064370). ADH2 protein is especially engaged in anaerobic processes in plants, while class III ADHs are also called glutathione-dependent formaldehyde dehydrogenase [66]. The proteome data correlated with the validation of ADH protein and *adh2* gene expression analysis, because the highest up-regulation of the *adh2* gene was observed on the first day of the first exposure for both accessions, although for WL-S this was almost two-fold higher than for WL-T. This is in agreement with the statement that the intensity of transcription and translation processes of marker genes for hypoxia, such as ADH, is at a similar level [12]. A greater abundance of ADH protein was noted previously in tomato roots after a one-time waterlogging [14]. For two peanut cultivars, a two-fold higher enzymatic activity for ADH was detected in the roots of the sensitive cultivar than for the tolerant cultivar [31]. After the second hypoxia exposure, *adh2* expression in the WL-S accession was similar as for WL-T, which could suggest the adjustment of the sensitive accession and a similar response to that of the tolerant one. In the present study, the qRT-PCR results correlate with the content level of ADH protein detected via Western blotting. Interestingly, for the control group, no presence of ADH protein was noted. This information is contrary to the statement that the alcohol fermentation pathway occurs also in mature plants grown under aerobic conditions, especially in roots [5]. However, the presented results of the accumulation of ADH agree with the report that hypoxia-sensitive plants show higher activity of enzymes of alcohol fermentation than tolerant ones [67]. Under anoxia in dryland plants, the accumulation of ethanol has been indicated to be more than twice higher than for wetland plants [68]. In the present research, the decrease in the *adh2* gene and ADH protein for WL-S after the second waterlogging suggested a higher tolerance of this genotype in comparison with the first waterlogging and acquisition of tolerance for hypoxia stress.

The presented results are partially in agreement with research on tolerant and sensitive accessions of cucumber under hypoxia conditions [25]. A higher level of *adh* gene expression was observed in the sensitive cucumber than for the tolerant one, after both the first and second waterlogging exposure. The adaptation to hypoxia conditions depends upon the species [69] and even specific genotype within the species [17]. Thus, the choice of accessions used in experiments could substantively influence the results, conclusions, and understanding of the mechanisms of hypoxia tolerance [69].

In the present study, the proteome showed a higher GAPDH protein expression in WL-S. Additionally, the proteome shows a similar up-regulation of GAPDH protein dedicated for a locus present on chromosome 5 (Solyc05g014470), as well as a locus located on chromosome 6 (Solyc06g071920). However, the validation of GAPDH protein expression was relatively similar to the control group for the WL-S and WL-T accessions after the first waterlogging. On the other hand, for both accessions a greater abundance of GAPDH was indicated at the beginning of the second hypoxia, which subsequently decreased; however, the expression was still higher than for the control. As was mentioned, the GAPDH protein is strongly connected with hypoxia stress as a key enzyme in the glycolytic pathway [1]. The significantly higher amount of GAPDH for both accessions under repeated hypoxia suggests that this enzyme could play a crucial role in adaptation to hypoxia stress through the increasing frequency of the glycolysis pathway. Anaerobic stress in rice causes an increase in GAPDH activity in the shoots and especially in the roots. Conversely, other research on rice seedlings indicates the *GAPDH* gene as a proposal for a reference gene with stable expression also under hypoxia conditions [70]. In the present study, a higher expression of the actin protein for WL-S after 1xH than for the control was observed. In plants, there are several isotypes of this ancient protein which play diverse roles [71]. The most common role is participation in cell division, organelle movement, vesicular trafficking, and cytoplasmic streaming. The *ACTIN* gene was the least stable of the set in *Chrysanthemum* under biotic and abiotic stress [72] and in rice under hypoxic conditions [70]. These data suggest that actin could be varied and modified in different species and tissues and could respond to stress conditions. Thus, actin’s role as a widely used reference gene should be tested especially in an experiment concerning the different stress conditions to ascertain its stability of expression [72]. These data are in accordance with our results, where the actin expression depended on the exposure. Moreover, in the present study, there was observed accumulation of ACT protein, which is coded on chromosome 3, and less abundance (fold change -3.1) of ACT protein coded on chromosome 10 (https://solgenomics.sgn.cornell.edu, accessed on 21 November 2021) for WL-S after 2xH. However, changes in protein expression level under different exposures, especially in WL-S, suggest the use of other reference genes than ACT for the analysis of gene expression under hypoxia stress in tomatoes.

### 4.4. Root Anatomy and ADH Detection Using Immunolabelling

In the present study, the roots showed an anatomy typical for dicotyledonous dryland plants with secondary growth [73]. The formation of aerenchyma was not detected either in the taproot or lateral roots. Some reports conducted especially on seedlings as early as a few decades ago have stated that cells from the meristematic and elongation zones of root tips are the first regions where the response to oxygen deficiency is observed [71,74]. One of the critical effects under long hypoxia exposure could be the death of meristematic cells due to a decrease in energy metabolism and cytoplasmic acidosis [74]. In the present study, the analysis was focused on the mature zone of the root, which shows lower critical values for respiration oxygen pressure than root apices [75].

Although there are many reports concerning the expression of *adh* genes or proteins in roots, stem or leaves, there are scarce data about their distribution in specific tissues and cells within an organ. Some attempts at the visualization of the expression of *adh* gene have done so with the use of the fusion with *beta-glucuronidase* (*gus*) or *green fluorescence protein* (*gfp*) reporter genes. However, the observations are mainly limited to a determination of which part of the plant shows positive staining, such as leaves, roots or root apex, which was reported for *Arabidopsis* plants under hypoxia or cold exposure [76]. This method revealed in rice that under anaerobic treatment the induction of the *adh* expression starts in the meristem of seedling roots [77]. 

Results from histochemical stains of *Petunia* seedlings during hypoxia show an increase in ADH activity in the root vasculature and then the spread of this activity through the cortex [78]. The vast majority of papers concerning dehydrogenase enzyme immunodetection in plants are focused on cinnamyl alcohol dehydrogenase (CAD), which is involved in the lignification pathway [79]. Therefore, in the present study, the results of ADH protein detection with the use of immunolabelling in the sections of mature taproots and secondary roots after the second waterlogging and hypoxia stress in tomatoes is a novelty.

It is known that the presence of the ADH enzyme, located in the cytoplasm and cytosol, is a marker of hypoxia stress [1,66]. In the present research, the histochemical analysis of ADH detection showed that the signals were observed for both accessions after hypoxia exposure. The presence of ADH was indicated in the cytoplasm of parenchyma cells of the cortex and vascular tissue, and also in cambium cells. In our study, the signal in the roots of control plants corresponds with the observed low amount of ADH protein. The alcohol fermentation pathway occurred also in mature plants grown under aerobic conditions, especially in roots rather than in shoots [5].

Vascular tissue is considered to be important in long-distance plant signaling [80], and one of the roles of parenchyma in xylem is carbon storage [81]. This could be an explanation for why the fluorescence signals were observed mainly within the parenchyma of vascular tissue rather than in the parenchyma of the cortex or pith. The parenchyma cells next to xylem and phloem could be the crucial place in the recognition of the deprivation of energy and the remodeling of metabolism under hypoxia exposure. More detailed immunohistochemical studies for the confirmation of this hypothesis are needed.

## 5. Conclusions

In the present study, distinct responses of sensitive and tolerant tomato accessions after the first and second waterlogging exposure at the level of protein expression were observed. The differences concerned, e.g., the proteins involved in metabolic pathways, especially carbon metabolism, enzymes engaged in oxidative stress, and also enzymes related to RNA molecule metabolism. Interestingly, changes in the abundance of proteins considered to be reference molecules, actin and tubulin, were noted. The response of WL-S tomatoes under the second hypoxia stress indicated an increase in tolerance connected with the limit of the ADH. Moreover, the up-regulation of some proteins related to various stress conditions (such as SNRK1, RBPs, PPIase, GLP, PLAT domain, wound/response protein, ASR protein and VDAC) in WL-S after 2xH suggested their possible role in hypoxia tolerance acquisition. Immunohistological analysis revealed the presence of ADH in the cytoplasm, mainly in the parenchyma cells of vascular tissue, the cortex, especially cells near the lateral roots, as well as in the epidermis and pith for both accessions. These observations indicated that cells of the different regions of roots were involved in the anaerobic metabolism. The present study showed that the proteome of primed plants was modified, and the priming exposure enhanced the tolerance of sensitive tomatoes to hypoxia stress, which could be important for crop yields. Additionally, these results broaden the knowledge about plants’ ability to adapt.

## Figures and Tables

**Table 1:** Significantly identified waterlogging-responsive tomato root spot proteins by MALDI-TOF/TOF MS analysis according to their metabolic function.

Spot ID	Protein Name	Funtional Annotation (Gene Ontology)	Protein Tomato Entry	Protein Fold Change (*p* < 0.01)					
				PZ 215 (WL-S)			POL 7/15 (WL-T)		
				1xH/Ctrl	Rec/ Ctrl	2xH/Ctrl	1xH/Ctrl	Rec/Ctrl	2xH/ Ctrl
***Exclusively in PZ 215:***									
3746	Endochitinase (Chitinase)	cell wall macromolecule catabolic process (GO:0016998)	Solyc10g055810	2.3	-	-	-	-	-
2065	Fructokinase 2	sucrose biosynthetic process (GO: 0005986)	Solyc06g073190	2.0	-	-	-	-	-
2215	Germin	response to cold (GO: 0009409), metal ion binding (GO: 0046872)	Solyc01g102380	1.7	-	-	-	-	-
3768	Cysteine proteinase cathepsin F	proteolysis (GO:0006508)	Solyc04g080960	−2.4	-	-	-	-	-
4667	Subtilisin-like protease	proteolysis (GO:0006508)	Solyc06g062950	−1.7	-	-	-	-	-
1261	Glutamine synthetase	nitrate assimilation (GO: 0042128)	Solyc04g014510	2.0	-	−2.9	-	-	-
4117	Heat shock protein	ATPase activity, coupled (GO:0042623), misfolded protein binding (GO:0051787)	Solyc03g082920	2.1	-	2.4	-	-	-
4477	Pathogenesis-related protein-1A1	defense response (GO:0006952)	Solyc01g106610	2.4	-	4.2	-	-	-
4526	Wound/stress protein	protein binding (GO:0005515)	Solyc03g096540	5.2	-	7.1	-	-	-
3404	S-adenosylmethionine synthase	*S-adenosylmethionine biosynthetic process (GO:0006556)*	Solyc12g099000	−1.5	-	−3.9	-	-	-
4272	Redoxin domain protein	cell redox homeostasis (GO:0045454)	Solyc01g079820	−1.6	-	−2.5	-	-	-
1439	Peroxidase 1	oxidoreductase activity (GO:0016491)	Solyc10g076220	−1.7	−1.6	-	-	-	-
3929	Germin-like protein	nutrient reservoir activity (GO:0045735)	Solyc01g102390	-	-	−2.1	-	-	-
***Exclusively in POL 7/15:***									
467	5-methyltetrahydropteroyltriglutamate -homocysteine methyltransferase	methionine biosynthetic process (GO:0009086)	Solyc10g081510	-	-	-	3.8	-	-
145	Alcohol dehydrogenase 2	alcohol dehydrogenase activity (GO:0004024)	Solyc06g059740	-	-	-	4.5	-	-
3473	Fructose-bisphosphate aldolase	glycolytic process (GO:0006096)	Solyc09g009260	-	-	-	3.7	-	-
4212	Pectinesterase	cell wall modification (GO:0042545)	Solyc02g080220	-	-	-	−1.6	-	-
4572	RNA binding protein-like protein	nucleic acid binding (GO:0003676)	Solyc05g053780	-	-	-	−1.7	-	−1.4
***Common in PZ 215 & POL 7/15:***									
3439	Alcohol dehydrogenase 2	alcohol dehydrogenase activity (GO:0004024)	Solyc06g059740	5.0	-	-	5.1	-	-
4134	Osmotin-like protein	protein binding (GO:0005515)	Solyc08g080670	9.4	-	-	4.7	-	-
4043	Triosephosphate isomerase	glycolytic process (GO:0006096)	Solyc04g011510	2.6	-	-	3.0	-	-
4019	Germin-like protein	nutrient reservoir activity (GO:0045735)	Solyc01g102400	2.6	-	1.6	2.4	1.4	-
4818	Alcohol dehydrogenase 2	alcohol dehydrogenase activity, zinc-dependent (GO:0004024)	Solyc06g059740	14.0	-	-	11.4	-	4.2
3418	Peroxidase	oxidoreductase activity (GO:0016491)	Solyc07g052510	−1.9	-	-	−1.7	-	-
3567	RNA-binding protein-like	mRNA binding (GO:0003729)	Solyc07g045240	−1.5	-	−3.0	-	-	−2.8
3351	UDP-D-glucose dehydrogenase	oxidation-reduction process (GO:0055114)	Solyc02g067080	−1.9	-	−12.3	-	-	−3.7
3378	Nuclear RNA binding protein	RNA binding (GO:0003723)	Solyc01g090190	-	-	−3.0	−1.7	-	−3.0
4072	Thaumatin-like protein	defense response (GO:0006952)	Solyc11g066130	-	-	−1.5	−2.1	-	−2.5
***Exclusively in PZ 215 2xH:***									
1796	Actin	ATP binding (GO:0005524)	Solyc03g078400	-	-	2.9	-	-	-
3892	Alcohol dehydrogenase 2	alcohol dehydrogenase activity (GO:0004024)	Solyc06g059740	-	-	5.3	-	-	-
3953	Coatomer subunit delta	endoplasmic reticulum to Golgi vesicle-mediated transport (GO:0006888)	Solyc01g103480	-	-	2.1	-	-	-
4768	Chitinase	cell wall macromolecule catabolic process (GO:0016998)	Solyc10g055800	-	-	3.7	-	-	-
4804	Embryo-specific 3	protein binding (GO:0005515)	Solyc03g116590	-	-	3.0	-	-	-
4760	Enolase	glycolytic process (GO:0006096)	Solyc09g009020	-	-	1.8	-	-	-
4533	FK506-binding protein 2	peptidyl-prolyl cis-trans isomerase activity (GO:0003755)	Solyc09g057670	-	-	1.6	-	-	-
4408	Glycine-rich RNA-binding protein	nucleic acid binding (GO:0003676)	Solyc10g051380	-	-	5.4	-	-	-
4209	Kunitz-type protease inhibitor	alpha-amylase inhibitor activity (GO:0015066)	Solyc03g019690	-	-	4.1	-	-	-
2552	Lipoxygenase homology domain-containing protein 1	catalase activity (GO:0004096)	Solyc04g054980	-	-	2.7	-	-	-
2474	Peptidyl-prolyl cis-trans isomerase	brassinosteroid signaling pathway (GO:0009742), response to cytokinin (GO:0009735)	Solyc01g111170	-	-	1.7	-	-	-
1294	Peroxidase 4	peroxidase activity (GO:0004601)	Solyc04g071890	-	-	1.8	-	-	-
2944	Porin/voltage-dependent anion-selective channel protein	transmembrane transport (GO:0055085)	Solyc02g067460	-	-	4.0	-	-	-
1106	S-adenosylmethionine synthase	S-adenosylmethionine biosynthetic process (GO:0006556)	Solyc09g008280	-	-	3.9	-	-	-
4347	SnRK1-interacting protein 1	group II intron splicing (GO:0000373)	Solyc08g005060	-	-	2.0	-	-	-
2511	Tubulin alpha-3 chain	microtubule cytoskeleton organization (GO:0000226)	Solyc02g091870	-	-	3.2	-	-	-
4482	Water-stress inducible protein 3	response to water deprivation (GO:0009414)	Solyc04g071610	-	-	6.3	-	-	-
4406	Wound/stress protein	protein binding (GO:0005515)	Solyc03g096540	-	-	3.1	-	-	-
4722	2 3-bisphosphoglycerate-independent phosphoglycerate mutase	carbohydrate metabolic process (GO:0044262)	Solyc07g044840	-	-	−10.1	-	-	-
3413	Actin	ATP binding (GO:0005524)	Solyc10g080500	-	-	−3.1	-	-	-
922	Adenosylhomocysteinase	S-adenosylmethionine cycle (GO:0033353)	Solyc09g092380	-	-	−3.3	-	-	-
954	Alanine aminotransferase	pyridoxal phosphate binding (GO:0030170)	Solyc03g123610	-	-	−2.2	-	-	-
980	Alcohol dehydrogenase 2	alcohol dehydrogenase activity (GO:0004024)	Solyc06g059740	-	-	−2.7	-	-	-
3949	Asparagine synthetase B	asparagine metabolic process (GO:0006528)	Solyc09g082780	-	-	−2.0	-	-	-
4766	ATP synthase subunit 1	ATP biosynthetic process (GO:0006754)	Solyc11g039980	-	-	−3.9	-	-	-
3287	ATP synthase subunit beta	ATP biosynthetic process (GO:0006754)	Solyc05g008460	-	-	−5.0	-	-	-
3529	Cathepsin B-like cysteine proteinase	proteolysis (GO:0006508)	Solyc12g088670	-	-	−1.6	-	-	-
3212	Chaperone DnaK	protein folding (GO:0006457)	Solyc01g106210	-	-	−3.0	-	-	-
4705	Chaperonin	protein folding (GO:0006457)	Solyc01g028810	-	-	−2.1	-	-	-
931	Acetyltransferase component of pyruvate dehydrogenase complex	pyruvate metabolic process (GO:0006090)	Solyc07g006790	-	-	−3.6	-	-	-
3583	Ferredoxin--NADP reductase	oxidation-reduction process (GO:0055114)	Solyc02g024050	-	-	−5.0	-	-	-
3601	Fructokinase 2	sucrose biosynthetic process (GO: 0005986)	Solyc06g073190	-	-	−3.6	-	-	-
3535	Fructose-bisphosphate aldolase	glycolytic process (GO:0006096)	Solyc05g008600	-	-	−2.9	-	-	-
3477	GDSL esterase/lipase	hydrolase activity (GO:0016788)	Solyc02g071700	-	-	−2.6	-	-	-
3423	Glutamate dehydrogenase	glutamate catabolic process (GO: 0006538)	Solyc10g078550	-	-	−2.9	-	-	-
3452	Glyceraldehyde 3-phosphate dehydrogenase	glycolytic process (GO:0006096)	Solyc05g014470	-	-	−4.6	-	-	-
1499	Guanine nucleotide-binding protein beta subunit-like protein	positive regulation of protein phosphorylation (GO:0001934)	Solyc12g040510	-	-	−2.2	-	-	-
3270	Leucyl aminopeptidase	proteolysis (GO:0006508)	Solyc12g010040	-	-	−3.8	-	-	-
3539	Malate dehydrogenase	carbohydrate metabolic process (GO:0005975)	Solyc09g090140	-	-	−3.8	-	-	-
3305	Mitochondrial processing peptidase alpha subunit	proteolysis (GO:0006508)	Solyc12g008630	-	-	−6.5	-	-	-
3604	Phenazine biosynthesis protein PhzF family	biosynthetic process (GO:0009058)	Solyc09g064940	-	-	−2.6	-	-	-
4702	Subtilisin-like protease	proteolysis (GO:0006508)	Solyc02g092670	-	-	−1.8	-	-	-
3208	Succinate dehydrogenase flavoprotein subunit	anaerobic respiration (GO: 0009061)	Solyc02g085350	-	-	−5.4	-	-	-
1023	Tubulin alpha-3 chain	microtubule cytoskeleton organization (GO:0000226)	Solyc04g077020	-	-	−7.2	-	-	-
3664	Tubulin beta chain	microtubule cytoskeleton organization (GO:0000226)	Solyc03g025730	-	-	−2.3	-	-	-
3291	UDP-D-glucose dehydrogenase	oxidation-reduction process (GO:0055114)	Solyc02g067080	-	-	−3.9	-	-	-
3341	UDP-glucosyltransferase	*secondary metabolite biosynthetic process (GO:0044550)*	Solyc11g066670	-	-	−3.4	-	-	-
991	UV excision repair protein RAD23	proteasome-mediated ubiquitin-dependent protein catabolic process (GO:0043161)	Solyc04g007120	-	-	−2.5	-	-	-
3321	V-type ATP synthase beta chain	ATP metabolic process (GO:0046034)	Solyc01g111760	-	-	−4.6	-	-	-
968	Xylose isomerase	carbohydrate metabolic process (GO:0005975)	Solyc07g006650	-	-	−2.7	-	-	-
4272	Redoxin domain protein	cell redox homeostasis (GO:0045454)	Solyc01g079820	−1.6	-	−2.5	-	-	-
3404	S-adenosylmethionine synthase	S-adenosylmethionine biosynthetic process (GO:0006556)	Solyc12g099000	−1.5	-	−3.9	-	-	-
3528	Adenosine kinase	purine ribonucleoside salvage (GO:0006166)	Solyc10g086190	-	−1.3	−2.8	-	-	-
4364	Class II heat shock protein	response to reactive oxygen species (GO:0000302)	Solyc08g062450	-	−1.5	−2.4	-	-	-
3590	Cysteine synthase	cysteine biosynthetic process from serine (GO:0006535)	Solyc09g082060	-	−2.4	−6.7	-	-	-
3298	Acetyltransferase component of pyruvate dehydrogenase complex	pyruvate metabolic process (GO:0006090)	Solyc07g006790	-	−2.3	−2.7	-	-	-
3242	Protein disulfide isomerase	proteolysis (GO:0006508)	Solyc06g005940	-	−1.8	−3.7	-	-	-
3337	Tubulin alpha-7 chain	microtubule cytoskeleton organization (GO:0000226)	Solyc02g087880	-	−2.7	−4.6	-	-	-
***Exclusively in POL 7/15 2xH:***									
1849	Alcohol dehydrogenase 2	alcohol dehydrogenase activity (GO:0004024)	Solyc06g059740	-	-	-	-	-	1.8
4839	Peptidyl-prolyl cis-trans isomerase	brassinosteroid signaling pathway (GO:0009742), response to cytokinin (GO:0009735)	Solyc01g111170	-	-	-	-	2.0	1.8
1185	Actin	ATP binding (GO:0005524)	Solyc11g005330	-	-	-	-	-	−2.8
4079	Adenylate kinase	pyrimidine nucleotide biosynthetic process (GO:0006221)	Solyc01g088480	-	-	-	-	-	−1.9
3959	Asparagine synthetase B	asparagine metabolic process (GO:0006528)	Solyc09g082780	-	-	-	-	-	−2.3
4197	Cold shock protein-1	heat shock protein binding (GO:0031072)	Solyc01g111300	-	-	-	-	-	−2.8
631	Heat shock protein	ATPase activity, coupled (GO:0042623), misfolded protein binding (GO:0051787)	Solyc09g010630	-	-	-	-	-	−2.1
908	Methylmalonate-semialdehyde dehydrogenase	oxidation-reduction process (GO:0055114)	Solyc01g106080	-	-	-	-	-	−1.7
3422	Patatin	phospholipase activity (GO:0004620)	Solyc08g006860	-	-	-	-	-	−2
4599	Peptidyl-prolyl cis-trans isomerase	chaperone-mediated protein folding (GO:0061077)	Solyc01g105710	-	-	-	-	-	−1.8
3576	Peroxidase 57	oxidation-reduction process (GO:0055114)	Solyc09g072700	-	-	-	-	-	−1.6
1575	Proteasome subunit alpha type	ubiquitin-dependent protein catabolic process (GO:0006511)	Solyc04g080590	-	-	-	-	-	−1.9
1188	Protein phosphatase 2C	phosphatase activity (GO:0016791)	Solyc03g007270	-	-	-	-	-	−1.9
3344	Reductase	oxidation-reduction process (GO:0055114)	Solyc08g081530	-	-	-	-	-	−2.8
3367	SGT1	protein binding (GO:0005515)	Solyc03g007670	-	-	-	-	-	−1.7
4821	Succinate dehydrogenase iron-sulfur	aerobic respiration (GO:0009060)	Solyc02g093680	-	-	-	-	-	−1.7
4355	Ubiquitin-conjugating enzyme family protein-like	ubiquitin conjugating enzyme binding (GO:0031624)	Solyc10g083120	-	-	-	-	-	−1.6
3748	V-type ATP synthase alpha chain	ATP metabolic process (GO:0046034)	Solyc12g055800	-	-	-	-	-	−2.2
***Common in PZ 215 & POL 7/15 (2xH):***									
530	Subtilisin-like protease	proteolysis (GO:0006508)	Solyc08g079870	-	-	6.0	-	-	4.4
3652	Peroxidase	oxidation-reduction process (GO:0055114)	Solyc01g105070	-	-	3.4	-	-	4.2
2662	Glyceraldehyde 3-phosphate dehydrogenase dehydrogenase	glycolytic process (GO:0006096)	Solyc05g014470	-	-	5.9	-	-	2.7
4467	Glyceraldehyde-3-phosphate dehydrogenase dehydrogenase	glycolytic process (GO:0006096)	Solyc06g071920	-	-	6.6	-	-	2.5
4559	Thiosulfate sulfurtransferase rhodanese domain protein	thiosulfate sulfurtransferase activity (GO:0004792)	Solyc02g083730	-	-	4.9	-	-	3.2
3934	Alcohol dehydrogenase 2	alcohol dehydrogenase activity (GO:0004024)	Solyc06g059740	3.7	1.8	8.0	-	-	6.7
2602	LH2 PLAT domain-containing protein	protein binding (GO:0005515)	Solyc03g096550	4.0	-	6.2	-	-	4.6
4514	Wound/stress protein	protein binding (GO:0005515)	Solyc03g096540	-	-	3.7	2.4	-	3.7
1138	Activator of heat shock protein ATPase homolog 1	chaperone binding (GO:0051087)	Solyc10g078930	-	-	−6.1	-	-	−3.2
669	Chaperone DnaK	protein folding (GO:0006457)	Solyc01g106210	-	-	−6.3	-	-	−3.8
3392	Cinnamyl alcohol dehydrogenase	oxidation-reduction process (GO:0055114)	Solyc01g107590	-	-	−6.5	-	-	−3.5
3340	Acetyltransferase component of pyruvate dehydrogenase complex	pyruvate metabolic process (GO:0006090)	Solyc07g006790	-	-	−3.4	-	-	−1.6
4723	Dihydroxy-acid dehydratase	branched-chain amino acid biosynthetic process (GO:0009082)	Solyc12g043020	-	-	−9.4	-	-	−3.4
4846	Glyceraldehyde 3-phosphate dehydrogenase	glycolytic process (GO:0006096)	Solyc05g014470	-	-	−2.8	-	-	−1.8
1024	Heterogeneous nuclear ribonucleoprotein A3	RNA binding (GO:0003723)	Solyc09g090520	-	-	−3.8	-	-	−2.2
3244	Ketol-acid reductoisomerase	branched-chain amino acid biosynthetic process (GO:0009082)	Solyc07g053280	-	-	−3.8	-	-	−2.6
3543	Malate dehydrogenase	carbohydrate metabolic process (GO:0005975)	Solyc09g090140	-	-	−4.3	-	-	−3.2
4728	Insulinase	proteolysis (GO:0006508)	Solyc02g088700	-	-	−4.5	-	-	−1.8
489	NADH dehydrogenase [ubiquinone] iron-sulfur protein 1	cellular respiration (GO:0045333)	Solyc11g011470	-	-	−6.0	-	-	−2.7
1030	Reductase	oxidation-reduction process (GO:0055114)	Solyc08g081530	-	-	−3.6	-	-	−1.8
3567	RNA-binding protein-like	mRNA binding (GO:0003729)	Solyc07g045240	−1.5	-	−3.0	-	-	−2.8
3412	S-(hydroxymethyl)glutathione dehydrogenase	ethanol oxidation (GO:0006069)	Solyc09g064370	-	-	−4.6	-	-	−2.1
622	Stress-induced protein sti1-like protein	protein binding (GO:0005515)	Solyc08g079170	-	-	−6.0	-	-	−2.5
714	Succinate dehydrogenase flavoprotein subunit	anaerobic respiration (GO: 0009061)	Solyc02g085350	-	-	−5.3	-	-	−2.6
3202	Transketolase 1	transketolase activity (GO:0004802)	Solyc05g050970	-	-	−15.0	-	-	−4.2
959	Tubulin beta chain	microtubule cytoskeleton organization (GO:0000226)	Solyc10g080940	-	-	−5.2	-	-	−3.3
3317	Tubulin beta-1 chain	microtubule cytoskeleton organization (GO:0000226)	Solyc04g081490	-	-	−5.6	-	-	−4.3
1063	UDP-glucose 6-dehydrogenase	oxidation-reduction process (GO:0055114)	Solyc02g088690	-	-	−5.0	-	-	−2.5
1022	UV excision repair protein RAD23	proteasome-mediated ubiquitin-dependent protein catabolic process (GO:0043161)	Solyc02g063130	-	-	−2.1	-	-	−2.2
3215	V-type ATP synthase alpha chain	ATP metabolic process (GO:0046034)	Solyc12g055800	-	-	−4.3	-	-	−3.0
2460	Eukaryotic translation IF 5A	translation (GO:0006412)	Solyc07g005560	-	-	−1.2	-	-	−2.4
4072	Thaumatin-like protein	defense response (GO:0006952)	Solyc11g066130	-	-	−1.5	−2.1	-	−2.5
***Exclusively in PZ 215 Rec:***									
1429	Fructokinase 2	sucrose biosynthetic process (GO: 0005986)	Solyc06g073190	-	−1.9	-	-	-	-
4364	Class II heat shock protein	response to reactive oxygen species (GO:0000302)	Solyc08g062450	-	−1.5	−2.4	-	-	-
3590	Cysteine synthase	cysteine biosynthetic process from serine (GO:0006535)	Solyc09g082060	-	−2.3	−6.7	-	-	-
3298	Acetyltransferase component of pyruvate dehydrogenase complex	pyruvate metabolic process (GO:0006090)	Solyc07g006790	-	−2.3	−2.7	-	-	-
3242	Protein disulfide isomerase	proteolysis (GO:0006508)	Solyc06g005940	-	−1.8	−3.7	-	-	-
3337	Tubulin alpha-7 chain	microtubule cytoskeleton organization (GO:0000226)	Solyc02g087880	-	−2.7	−4.6	-	-	-
3236	2,3-bisphosphoglycerate-independent phosphoglycerate mutase	carbohydrate metabolic process (GO:0044262)	Solyc07g044840	-	−1.6	-	-	-	−2.2
3351	UDP-D-glucose dehydrogenase	oxidation-reduction process (GO:0055114)	Solyc02g067080	-	−1.9	−12.3	-	-	−3.7
***Exclusively in POL 7/15 Rec:***									
3825	Actin	ATP binding (GO:0005524)	Solyc02g067080	-	-	-	-	1.1	-
4130	Osmotin-like protein	protein binding (GO:0005515)	Solyc08g080660	-	-	-	-	1.5	-
4260	Tubulin alpha-3 chain	microtubule cytoskeleton organization (GO:0000226)	Solyc04g077020	-	-	-	-	1.6	-
4839	Peptidyl-prolyl cis-trans isomerase	brassinosteroid signaling pathway (GO:0009742), response to cytokinin (GO:0009735)	Solyc01g111170	-	-	-	-	2.0	1.8
1213	Peroxidase	oxidoreductase activity (GO:0016491)	Solyc07g052510	-	-	-	-	−1.8	-
4697	Peroxidase 4	oxidoreductase activity (GO:0016491)	Solyc04g071890	-	-	-	-	−2.2	-
726	Pyruvate decarboxylase	pyruvate decarboxylase activity (GO:0004737)	Solyc10g076510	-	-	-	-	−1.5	-

Red and blue colours indicate direction of protein fold change in relation to the control (Ctrl) (red—more abundance, blue—less abundance).

## Data Availability

Data are contained within the article or supplementary material.

## Supplementary Materials

The following are available online at https://www.mdpi.com/article/10.3390/cells11030500/s1, Supplementary Table S1: The differences in morphological and physiological characteristics in response to waterlogging stress in two tomato accessions, Supplementary Table S2: Experimental design for protein labeling, Supplementary Table S3: Correlationships between PCs and the different quantitative variable spots, Supplementary Figure S1: Pictures of tomato plants under hypoxia stress, Supplementary Figure S2: 2D-DIGE map of differential abundance proteins, Supplementary Figure S3. Steedman’s Wax embedded tomato cross sections of leaves and roots, Supplementary Figure S4: Steedman’s Wax embedded tomato cross sections of PZ 215 and POL 7/15 accessions taproot and lateral roots after the first (1xH) and second (2xH) waterlogging, Supplementary Data S1: Identified proteins from each fraction and their abundance change due to hypoxia stress exposure, Supplementary Data S2: Complete MASCOT protein identification data of picked spots.
