# Supplementary material for: Proteomic Studies of Roots in Hypoxia-Sensitive and -Tolerant Tomato Accessions Reveal Candidate Proteins Associated with Stress Priming
Source: Cells. 2022 Jan 31;11(3):500. doi: 10.3390/cells11030500 (PMC8834170; doi:10.3390/cells11030500)
Supplement: Supplementary file 1 [file cells-11-00500-s001.zip › cells-1515865-sp/cells-1515865-sp-conversion.pdf]

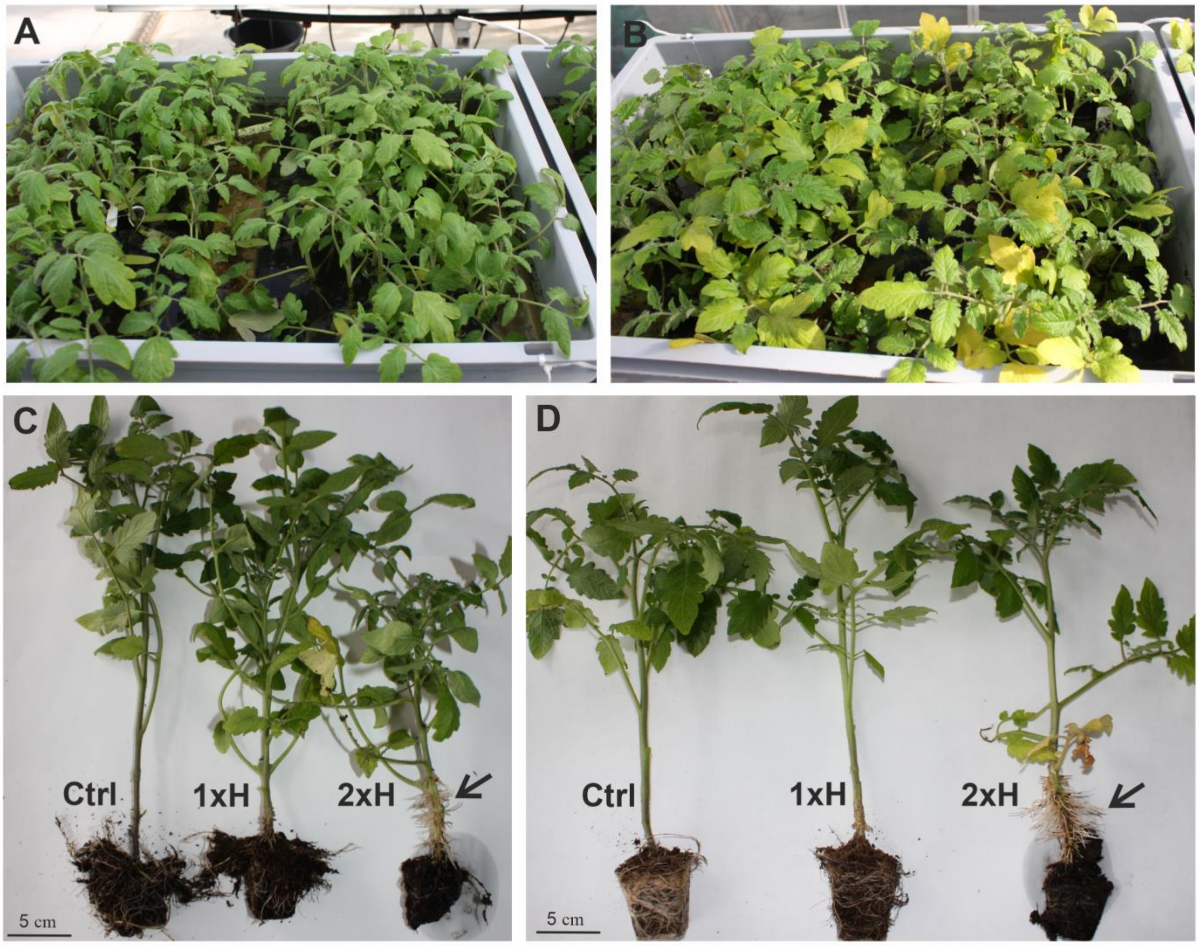

Suplementary Figure S1. The plants of POL 7/15 (A) and PZ 215 (B) tomato accessions which were exposed to hypoxia stress for 7 days by waterlogging (1xH). After that, plants were taken out of the water and stayed unstressed for 14 days (Rec). Next, plants from the 1xH group were waterlogged for the second time for another 7 days (2xH). At 28 day of the experiment control plants (Ctrl), plants after first (1xH) and second (2xH) waterlogging of POL 7/15 and PZ 215 tomato accessions are presented in the pictures C and D, respectively. The arrow marks the adventitious roots growing on the shoot under the waterlogging stress.

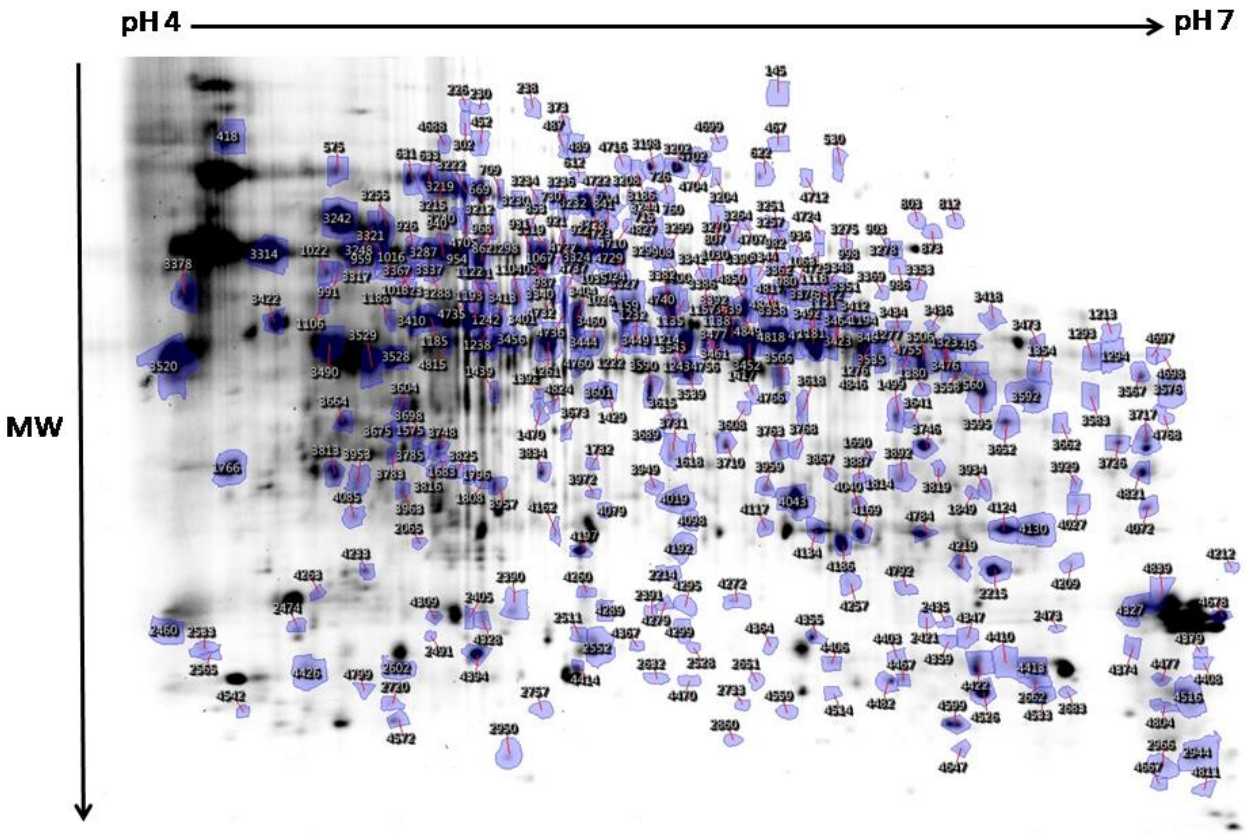

Suplementary Figure S2. 2D-DIGE map of differential protein abundance analysis. Representative 2D-DIGE gel image showing the spot map corresponding to the internal standard (Cy2 labeling), which is common to all gels analyzed. Sample preparation and labeling were performed as described in the Material and Methods section. Indicated spots were selected for picking based on statistical parameters calculated by the SameSpots software They showed a statistically significant variation of spot volume with 99% confidence level ($P < 0.01$). These spots were removed for subsequent protein identification by mass spektrometry and they are listed as Supplementary Data 1

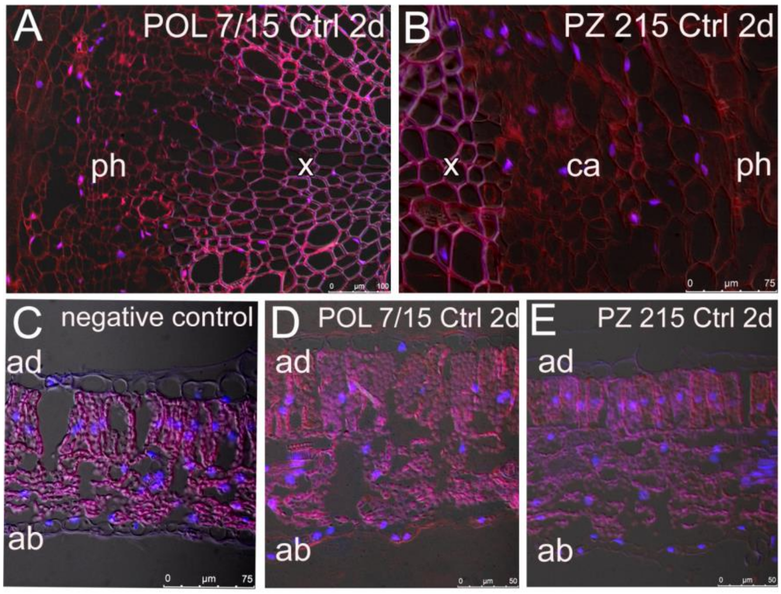

Supplementary Figure S3. Steedman's Wax embedded cross sections of leaves and roots of *Solanum lycopersicum*. A and D Root and leaf tissue of POL 7/15 accession control plants after 2 days; the signal in all of the tissue (i.e. phloem, *ph* or xylem, *x*) of taproot and chloroplasts in palisade mesophyll cells on the adaxial side of the leaf (*ad*), and in the spongy mesophyll on the abaxial side of the leaf (*ab*). C Leaf tissue labelled omitting the primary antibody; lack of signal in all of the tissue (i.e. cortex cells, *co* apart from autofluorescent signal of xylem, *x* of lateral roots *LR* ,and chloroplasts in palisade mesophyll cells on the adaxial side of the leaf (*ad*), and in the spongy mesophyll on the abaxial side of the leaf (*ab*). B and E Root and leaf tissue of PZ 215 accession control plants after 2 days; the signal in all of the tissue (i.e. phloem, *ph*; cambium, *ca* or parenchymal cells of xylem, *x*) of taproot and chloroplasts in palisade mesophyll cells on the adaxial side of the leaf (*ad*), and in the spongy mesophyll on the abaxial side of the leaf (*ab*). Scale bar = 100 µm (A), 75 µm (B, C), 50 µm (D, E)

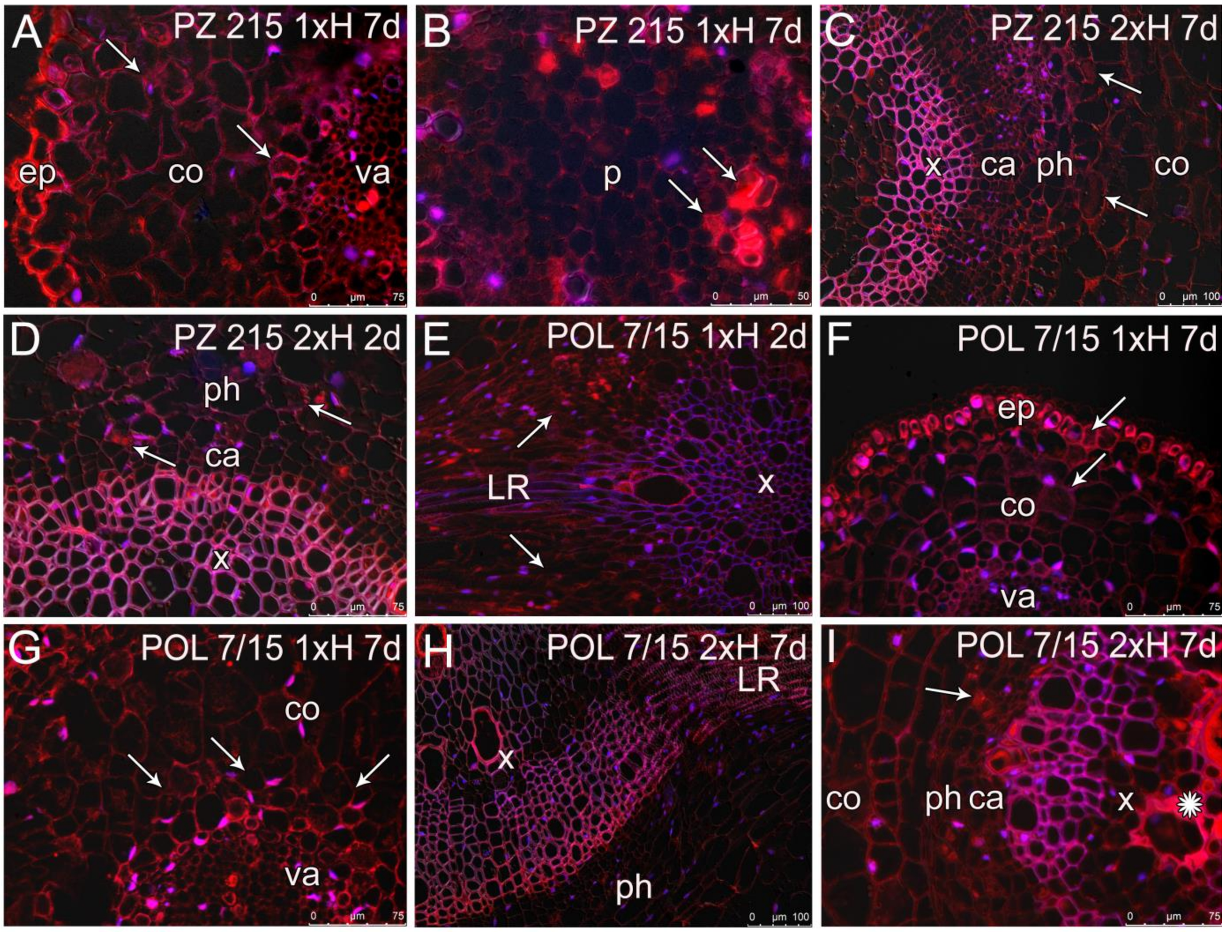

Supplementary Figure S4. Steedman's Wax embedded cross sections of the *Solanum lycopersicum* PZ 215 accession taproot and lateral roots after the first (1xH, A, B) and second (2xH, C, D) waterlogging, and POL 7/15 accession tap root and lateral roots after the first (1xH, E – G) and second (2xH, H – I) waterlogging. A: The ADH signal in lateral roots is mainly noted in the cytoplasm of cortex cells (co) and in vascular tissues (va), as well as in epidermal cells (ep). B: A magnification of the details from C; parenchymal cells of pith (*pt*) with an accumulation of ADH in a few cells (*arrows*). C and D: ADH activity labelled mainly in phloem cells (*ph*) in the taproot and cortex cells (*arrows*); the signal detected in cambium (*ca*) or parenchymal cells of xylem (*x*). E: Part of the stem of the taproot with lateral root (*LR*) formation; the ADH signal observed in vascular tissues: phloem (*ph, arrows*) and in parenchymal cells of xylem (*x*), cambium (*ca*), surrounding cortex cells (*co*) of the taproot. F The ADH signal in lateral roots is mainly noted (*arrows*) in the cytoplasm of cortex cells (*co*) and in epidermal cells (*ep*), the signal labelled in vascular tissue (*va*). G A magnification of the details from F. H and I The signal of ADH detected only in a few cells of phloem (*ph, arrow*) and a few parenchymal cells of the xylem (*x, arrow*); a near absence of signal detected in xylem (*x*) cambium (*ca*) and cortex cells (*co*) of lateral roots; autofluorescence of vascular elements which represent a strong signal are marked by *asterisk*. Scale bar = 100 µm (C, E, H), 75 µm (A, D, F, G, I), 50 µm (B)

Supplementary Table S1. Mean value of 18 mophological and physiological parameters estimated in control and plants exposed to 7 days-hypoxia stress of two tomato accessions POL 7/15 and PZ 215. Bold p-values and indicated cells in gray indicate statistically significant differences between control and stressed plants of each accession separately estimated with Student's t-test and $p < 0.05$. According to published data by Kołton et al. (2020) [17].

Parameter	POL 7/15 Control	POL 7/15 Stress	p-Value	PZ 215 Control	PZ 215 Stress	p-Value
% weight change	100	117	**0.0005**	100	109	**0.0332**
% height change	100	70	**0.0239**	100	11	**0.0000**
Relative leaf number	1.10	1.05	0.8010	0.53	0.16	**0.0160**
F_0	441	447	0.6030	456	677	**0.0069**
Fm	2259	2317	0.5088	2371	2211	0.0645
Fv	1818	1869	0.5695	1915	1534	**0.0053**
Fv/F_0	4.17	4.20	0.9211	4.22	2.82	**0.0014**
Fv/Fm	0.80	0.81	0.5391	0.81	0.68	**0.0034**
Tfm	178	191	0.3619	183	244	**0.0000**
Area	19,874	20,181	0.8558	20,171	13,332	**0.0010**
Fm/F_0	5.17	5.20	0.9215	5.22	3.82	**0.0014**
PI ABS	1.13	0.95	0.3583	0.97	0.43	**0.0008**
ABS/RC	3.15	3.18	0.8164	3.20	5.13	**0.0009**
TR_0/RC	2.51	2.56	0.3718	2.58	3.22	**0.0000**
ET_0/RC	1.04	1.05	0.8469	1.04	0.97	0.3251
DI_0/RC	0.65	0.62	0.6056	0.62	1.91	**0.0051**
ET_0/ABS	0.34	0.33	0.7765	0.33	0.23	**0.0019**
ET_0/TR_0	0.42	0.41	0.7011	0.41	0.31	**0.0036**

Supplementary Table S2. Experimental design for protein labeling. Pool served as an internal standard and consisted of equal amounts of each sample analyzed in 2D-DIGE.

Gel number	Cy2-labeled	Cy3-labeled	Cy5-labeled
Gel 1	Pool	PZ 215 Ctrl-1	PZ 215 2xH-39
Gel 2	Pool	PZ 215 Ctrl-3	PZ 215 1xH-17
Gel 3	Pool	POL 7/15 Ctrl-9	POL 7/15 Rec-36
Gel 4	Pool	PZ 215 1xH-15	POL 7/15 Ctrl-12
Gel 5	Pool	POL 7/15 1xH-19	POL 7/15 2xH-48
Gel 6	Pool	POL 7/15 1xH-23	PZ 215 Rec-30
Gel 7	Pool	PZ 215 Rec-27	PZ 215 2xH-38
Gel 8	Pool	PZ 215 Rec-28	POL 7/15 Rec-35
Gel 9	Pool	POL 7/15 Rec-33	POL 7/15 1xH-24
Gel 10	Pool	PZ 215 2xH-37	PZ 215 1xH-16
Gel 11	Pool	POL 7/15 2xH-43	POL 7/15 Ctrl-11
Gel 12	Pool	POL 7/15 2xH-45	PZ 215 Ctrl-56

Supplementary Table S3. Correlationships beetween PCs and the different quantitative variable spots. Protein spot abundance clustering based on PCA. For each PC, two clusters of proteins were identified that were positively and negatively correlated with the PC. The degree of protein abundance change within a specific PC was measured by the slope of regression of log-transformed protein abundance versus the corresponding eigenvector multiplied by the range of values within the eigenvector. If the degree of protein abundance change exceeded the onefold change threshold, the protein spot was considered to be associated with the PC. Protein clustering was performed sequentially starting from the first PC. Proteins that were already clustered with a PC were not included in the clusters associated with subsequent PCs and were presented as **Figure 2E and Figure 2F** for PZ 215 and POL 7/15 tomato accessions, respectively.

Tomato accession	PC number	Direction	# Spot	Protein name	LogChange	Correlation
**PZ 215**	PC1	Positive	4482	Water-stress inducible protein 3	0.703	0.725
			4792	S-adenosylmethionine synthase	0.537	0.811
		Negative	4784	Osmotin-like protein	-0.482	-0.703
	PC2	Negative	530	Subtilisin-like protease	-0.709	-0.821
			1106	S-adenosylmethionine synthase	-0.508	-0.810
			1294	Peroxidase 4	-0.310	-0.860
			1796	Actin	-0.363	-0.733
			2602	Wound/stress protein	-0.789	-0.992
			2662	Glyceraldehyde 3-phosphate dehydrogenase	-0.692	-0.927
			2944	Porin/voltage-dependent anion-selective channel protein	-0.487	-0.813
			3892	Alcohol dehydrogenase 2	-0.664	-0.900
			3934	Alcohol dehydrogenase 2	-0.862	-0.998
			4117	Heat shock protein	-0.374	-0.966
			4209	Kunitz-type protease inhibitor	-0.553	-0.945
			4327	Chitinase	-0.342	-0.820
			4406	Wound/stress protein	-0.404	-0.873
			4408	Glycine-rich RNA-binding protein	-0.611	-0.754
			4467	Glyceraldehyde-3-phosphate dehydrogenase	-0.688	-0.876
			4477	Pathogenesis-related protein-1	-0.584	-0.981
			4514	Wound/stress protein	-0.519	-0.961
			4526	Wound/stress protein	-0.833	-0.970
			4559	Thiosulfate sulfurtransferase rhodanese domain protein	-0.751	-0.968
			4768	Chitinase	-0.459	-0.834

**POL 7/15**	PC1	Positive	4839	Peptidyl-prolyl cis-trans isomerase	0.335	0.775
		Negative	3446	Nuclear RNA binding protein	-0.565	-0.942
			3566	Alcohol dehydrogenase 2	-0.836	-0.926
			4559	Thiosulfate sulfurtransferase rhodanese domain protein	-0.557	-0.775
	PC2	Negative	530	Subtilisin-like protease	-0.539	-0.812
			3652	Peroxidase	-0.504	-0.867
			3934	Alcohol dehydrogenase 2	-0.599	-0.775
	PC3	Negative	2602	Wound/stress protein	-0.456	-0.902
			2662	Glyceraldehyde 3-phosphate dehydrogenase	-0.465	-0.974
			4134	Osmotin-like protein	-0.562	-0.827
			4467	Glyceraldehyde-3-phosphate dehydrogenase	-0.377	-0.796
			4514	Wound/stress protein	-0.442	-0.833
